# Redefining COVID-19 Severity and Prognosis: The Role of Clinical and Immunobiotypes

**DOI:** 10.3389/fimmu.2021.689966

**Published:** 2021-09-08

**Authors:** Jiram Torres-Ruiz, Alfredo Pérez-Fragoso, José Luis Maravillas-Montero, Luis Llorente, Nancy R. Mejía-Domínguez, José Carlos Páez-Franco, Sandra Romero-Ramírez, Victor Andrés Sosa-Hernández, Rodrigo Cervantes-Díaz, Abdiel Absalón-Aguilar, Miroslava Nuñez-Aguirre, Guillermo Juárez-Vega, David Meza-Sánchez, Ari Kleinberg-Bid, Thierry Hernández-Gilsoul, Alfredo Ponce-de-León, Diana Gómez-Martín

**Affiliations:** ^1^Department of Immunology and Rheumatology, Instituto Nacional de Ciencias Médicas y Nutrición Salvador Zubirán, Mexico City, Mexico; ^2^Emergency Medicine Department, Instituto Nacional de Ciencias Médicas y Nutrición Salvador Zubirán, Mexico City, Mexico; ^3^Red de Apoyo a la Investigación, Universidad Nacional Autónoma de México e Instituto Nacional de Ciencias Médicas y Nutrición Salvador Zubirán, Mexico City, Mexico; ^4^Department of Infectious Diseases and Microbiology, Instituto Nacional de Ciencias Médicas y Nutrición Salvador Zubirán, Mexico City, Mexico

**Keywords:** COVID-19, TRIM63, NETs, LDGs, metabolomics, T cells, MCP-1, IP-10

## Abstract

**Background:**

Most of the explanatory and prognostic models of COVID-19 lack of a comprehensive assessment of the wide COVID-19 spectrum of abnormalities. The aim of this study was to unveil novel biological features to explain COVID-19 severity and prognosis (death and disease progression).

**Methods:**

A predictive model for COVID-19 severity in 121 patients was constructed by ordinal logistic regression calculating odds ratio (OR) with 95% confidence intervals (95% CI) for a set of clinical, immunological, metabolomic, and other biological traits. The accuracy and calibration of the model was tested with the area under the curve (AUC), Somer’s D, and calibration plot. Hazard ratios with 95% CI for adverse outcomes were calculated with a Cox proportional-hazards model.

**Results:**

The explanatory variables for COVID-19 severity were the body mass index (BMI), hemoglobin, albumin, 3-Hydroxyisovaleric acid, CD8+ effector memory T cells, Th1 cells, low-density granulocytes, monocyte chemoattractant protein-1, plasma TRIM63, and circulating neutrophil extracellular traps. The model showed an outstanding performance with an optimism-adjusted AUC of 0.999, and Somer’s D of 0.999. The predictive variables for adverse outcomes in COVID-19 were severe and critical disease diagnosis, BMI, lactate dehydrogenase, Troponin I, neutrophil/lymphocyte ratio, serum levels of IP-10, malic acid, 3, 4 di-hydroxybutanoic acid, citric acid, myoinositol, and cystine.

**Conclusions:**

Herein, we unveil novel immunological and metabolomic features associated with COVID-19 severity and prognosis. Our models encompass the interplay among innate and adaptive immunity, inflammation-induced muscle atrophy and hypoxia as the main drivers of COVID-19 severity.

## Introduction

The novel beta coronavirus severe acute respiratory syndrome coronavirus 2 (SARS-CoV-2) emerged in December 2019 in Wuhan, China and is the cause of the coronavirus disease 2019 (COVID-19) (1), that was acknowledged by the World Health Organization as a pandemic in March 2020 (2), and has challenged and overwhelmed all the healthcare systems around the world (3).

Currently, the American continent is the epicenter of this pandemic, contributing with 51% of all new cases and 45% of all new deaths as reported in the first week of 2021 (4). Latin American (LA) countries, in particular, are being devastated by this disease, and information regarding the risk factors of COVID-19 severity in LA is quite scant. In Mexico, as of January 20, 2021, there have been approximately 1,600,000 confirmed cases and more than 142,000 deaths (5). Beyond the available number of beds with ventilators, concerns about the quality of care have also been raised, as suggested by the high mortality rate for intubated COVID-19 patients in Mexico, compared with other countries (6).

Current evidence suggests that besides the clinical risk factors related to COVID-19 severity, there are diverse immunological (7, 8), metabolic (9) and muscle (10) abnormalities that also play a key role in disease severity. Although diverse prognostic models for COVID-19 severity have been proposed, most of them only include limited immunological variables, with clinical, radiographic and laboratory features, without the incorporation of a comprehensive assessment of the wide COVID-19 spectrum of abnormalities. Besides, most prognostic models for adverse outcomes are based on Asian and European populations (11–14). Therefore, the aim of the present study was to create and validate a compound explanatory model including diverse clinical, immunological, metabolomic, and muscle atrophy variables to classify COVID-19 patients according to their disease severity and to predict adverse outcomes.

## Material and Methods

We recruited a cohort of 121 patients with COVID-19 confirmed by a positive polymerase chain reaction (PCR) for SARS-CoV-2 in nasopharyngeal swab who consecutively attended to the emergency department of the Instituto Nacional de Ciencias Médicas y Nutrición Salvador Zubirán, a reference hospital for patients with COVID-19 in Mexico from March to August, 2020. This study was approved by the institutional Ethics and Research committees (REF: 3341) according to the Helsinki declaration and all patients signed a written informed consent prior to their inclusion.

At hospital admission, patients had an exhaustive medical approach including a complete medical history and a low radiation high-definition non-contrasted thoracic computed tomography (CT). Prior to the initiation of medical treatment, a blood sample was drawn for the assessment of the following laboratory tests: complete blood count, glucose, blood urea nitrogen (BUN), creatinine, liver function tests, ultra-sensitive C-reactive protein (CRP), lactate dehydrogenase (LDH), creatine phosphokinase (CPK), troponin-I, thromboplastin time (TP), activated partial thromboplastin time (aPTT), D-dimer, fibrinogen, and arterial blood gases. All plasma and serum samples were stored at -80°C until further analysis. We calculated the following pneumonia severity scores: Pneumonia Severity Index/Pneumonia Patient Outcomes Research Team (PSI/PORT), CURB-65 (confusion, urea, respiratory rate, blood pressure and age ≥65), National Early Warning Score (NEWS) 2, quick Sequential Organ Failure Assessment score (qSOFA), SMART-COP (systolic blood pressure, multilobar chest radiography involvement, albumin level, respiratory rate, tachycardia, confusion, oxygenation, and arterial pH) and MuLBSTA (multilobular involvement, absolute lymphocyte count, bacterial co-infection, smoking history, history of hypertension and age ≥60) (15). Patients were stratified according to their disease severity as following (16):

1. Mild/moderate disease: Fever, upper respiratory infection symptoms, with or without pneumonia.
2. Severe: Any of the following: respiratory failure, respiratory rate ≥ 30 breaths per minute, oxygen saturation at rest ≤ 93%, PaO_2_/FiO_2_ ≤ 300 mmHg.
3. Critical: Any of the following: requirement of invasive mechanical ventilation (IMV), shock, multiple organ failure.

Additionally, the following experimental procedures were performed:

### Multiparametric Flow Cytometry Characterization of Peripheral Blood Mononuclear Cells (PBMCs)

We isolated peripheral blood mononuclear cells (PBMCs) by density gradients after centrifugation with Ficoll-Paque (GE Healthcare Life Sciences, Illinois, USA). After washing the cells twice with phosphate buffered saline (PBS), we stained them with the viability marker Zombie Aqua (Biolegend, California, USA). The cells were washed twice with 5% fetal bovine serum (FBS) in PBS and incubated during 30 min at room temperature with the FcX blocker (Biolegend, California, USA) and the following fluorochrome-coupled antibodies: CD19-BUV496 (Cat. 612938, BD Biosciences, Franklin Lakes, New Jersey, USA), CD3-APC/Fire-750 (Cat. 344840), CD4-Alexa fluor 488 (Cat. 317420), CD8-PE/Dazzle-594 (Cat. 344744), CD10-PE (Cat. 312204), CD11c-PE/Dazzle-594 (Cat. 337228), CD14-PerCP (Cat. 325632), CD15-FITC (Cat. 301904), CD16-Alexa fluor 700 (Cat. 302026), CD21-Alexa fluor 700 (Cat. 354918), CD24-BV421 (Cat. 311122), CD25-BV421 (Cat. 302630), CD27-APC-Cy7 (Cat. 356424), CD38-BV650 (Cat. 356620), CD45RA-PE (Cat. 304108), CD45RO-FITC (Cat. 304242), CD56-PE (Cat. 318306), CD62L-PE/Cy5 (Cat. 304808), CD127-BV650 (Cat. 351326), CD335-BV650 (Cat. 331927), CD355-APC (339108), CCR7-PE/Dazzle-594 (Cat. 353236), IgD-PerCP/Cy5.5 (Cat. 405710) (all from Biolegend, California, USA). For the assessment of the T helper and cytotoxic subsets, we stimulated the PBMCs with phorbol 12-myristate 13-acetate (PMA), ionomycin and monensin during 5 hrs at 37°C. The cells were fixed and permeabilized with the cytofix/cytoperm fixation/permeabilization kit (BD Biosciences, New Jersey, USA) according to the instructions of the manufacturer. Intracytoplasmic cytokines were detected with the following fluorochrome-coupled antibodies: IFN-γ-APC (Cat. 506510), IL-4-PE (Cat. 500810), IL-17-BV421 (Cat. 512322) (all from Biolegend, California, USA). One million events were acquired in a 4-laser LSR Fortessa flow cytometer (BD Biosciences, New Jersey, USA). We characterized the PBMCs subsets depicted in **Supplementary Table 1**.

The gating strategy for every cell subset is depicted in **Supplementary Figures 1, 2**. The absolute number (cells/mcl) of LDG, monocyte and lymphocyte subsets were calculated according to the amount of total leukocytes, monocytes and lymphocytes respectively, in a complete blood count drawn at the same day of the obtention of PBMCs.

### Measurement of the Cytokine/Chemokine and Coagulation Profiles

The serum concentration of 32 cytokines and chemokines and 4 coagulation factors in plasma were measured using the MILLIPLEX Multi-Analyte Profiling (MAP) Human Cytokine/Chemokine Magnetic Bead Panel 29-plex kit (EMD Millipore, Darmstadt, Germany), the TGF-β Base Magnetic Luminex Performance Assay 3-plex kit (R&D Systems, Minneapolis, USA), and the ProcartaPlex Multiplex Immunoassay Human Coagulation Panel 4-plex kit (Thermo Fisher Scientific, Massachusetts, USA), on a 2-laser Bio-Plex 200 suspension array system coupled to a Bio-Plex Pro Wash Station (Bio-Rad, California, USA), according to the instructions of the manufacturers. Bead-fluorescence intensity readings for all the samples and standards were converted into the corresponding analyte concentrations using the Bio-Plex Manager software v6.2 (Bio-Rad, California, USA).

Analytes measured included: interleukin 1-α (IL-1α), IL-1β, IL-1RA, IL-2, IL-3, IL-4, IL-5, IL-6, IL-7, IL-8, IL-10, IL-12p40, IL-12p70, IL-13, IL-15, IL-17A, interferon α-2 (IFNα2), IFNγ, tumor necrosis factor-α (TNF-α), TNF-β, monocyte chemoattractant protein 1 (MCP-1)/CCL2, macrophage inflammatory protein 1-α (MIP-1α)/CCL3, macrophage inflammatory protein-1β (MIP-1β)/CCL4, interferon γ-induced protein (IP-10)/CXCL10, eotaxin-1/CCL11, epidermal growth factor (EGF), granulocyte colony-stimulating factor (G-CSF), granulocyte-macrophage colony-stimulating factor (GM-CSF), vascular endothelial growth factor (VEGF), transforming growth factor-β1 (TGF-β1), TGF-β2, TGF-β3, Factor IX, Protein C (Factor XIX), Protein S, and von Willebrand Factor (vWF). The serum levels of IL-18 were assessed by ELISA (MBL, Massachusetts, USA) according to the provider instructions.

### Appraisal of the Metabolomic Signature

We performed untargeted metabolomics analysis of sera from all patients employing gas chromatography coupled to mass spectrometry (GC/MS). Our metabolomic method detected 46 metabolites with relative standard deviation (RSD) <30% in the quality control (QC) sample, that consists of equal volumes of all the samples included in the analysis, as previously described (17). The detailed procedure is described in the **Supplementary Material**.

### Assessment of the Circulating Neutrophil Extracellular Traps (NETs)

The amount of plasma NETs was addressed by ELISA, as previously described (18). Briefly, high binding 96 well plates were coated overnight at 4°C with mouse anti human neutrophil elastase (NE) 1:2000 (Cat 481001, Calbiochem, Darmstadt, Germany) in coating buffer from the cell death detection ELISA kit (Roche, Basilea, Switzerland). We washed the plates three times with PBS/Tween20 and blocked the non-specific binding sites with 1% bovine serum albumin (BSA) in PBS for 6 hrs at room temperature for the detection of DNA-NE complexes as previously described (18). The plasma samples were diluted 1:10 in 1% BSA and incubated overnight at 4°C. After washing three times with PBS/Tween 20, we incubated the plates with the anti-human DNA-POD antibody from the cell death detection ELISA kit (Roche, Basilea, Switzerland). We washed the plate five times with PBS/Tween 20 and applied the TMB substrate (Thermofisher Scientific, Massachusetts, USA). The plate was read at 450 nm after applying stop solution and the optic density index (ODI) was calculated as previously described (18).

### Evaluation of the Markers of Muscle Atrophy, Hypoxia, Anti-Viral Response, and Oxidative Stress

We assessed by ELISA, following the provider instructions, the plasma levels of TRIM63 (MyBioSource, California, USA) and atrogin-1 (MyBioSource, California, USA) as markers of muscle atrophy. The serum concentrations of HIF-1α (Thermofisher Scientific, Massachusetts, USA) and 8-hydroxy 2 deoxyguanosine (Abcam, Cambridge, UK) to address tissue hypoxia and oxidative stress, and the plasma levels of TRIM21 (MyBioSource, California, USA) as part of the antiviral innate immune response were measured by a commercial ELISA as well.

Noteworthy, all the samples were processed by investigators blinded to the COVID-19 severity and outcome of each included subject.

### Statistics

Quantitative variables were expressed as medians and interquartile ranges (IQR). Differences among groups were assessed by the Kruskal-Wallis test. The sample size was not initially calculated because there was no available data about the size of the effect of the novel variables included in the COVID-19 severity predictive index. Nonetheless, the included sample size gave us enough power to detect statistically significant differences among groups. To address the factors associated with COVID-19 severity, an ordinal logistic regression model was developed including all the variables described above. First, we evaluated the correlation between variables and removed from the univariate analysis those with redundant statistical and biological information. Also, we discarded the clinical variables included in the definition of COVID-19 severity to avoid bias. We used a random forest trained algorithm on the observed values of a data matrix to predict the missing values (19). The outcome measured by the predictive model was COVID-19 severity, as mild/moderate, severe and critical (16).

To select the variables for the construction of the explanatory model for COVID-19 severity, we performed a univariate ordinal logistic regression. The statistically significant variables (P<0.05) were selected as candidate predictors. Finally, we chose the significant variables in the best fitting multivariate model using the minimum Akaike information criteria (AIC). Assumption of proportionality odds was verified with the Brant and Hosmer-Lemeshow tests.

Model performance and internal calibration were evaluated with conventional discrimination indexes: area under the curve (AUC, C-statistic), Somer’s D, Spearman’s rho, R^2^, and optimism-corrected overfitting. Calibration plot and discrimination indexes were obtained by bootstrapping 1000 samples of the original data. Accuracy metrics [sensitivity, specificity, positive predictive value (PPV), negative predictive value (NPV), positive likelihood ratio (+LR), and negative likelihood ratio (–LR)] were calculated by cross-validation and the goodness of fit of the model was assessed by Lipsitz test (20). Furthermore, we constructed a heat map by hierarchical clustering analysis with Ward’s method for visualization of the relationship between patient’s condition and the variables included in the index. Cox regression was used to assess the composite outcome of disease progression and death. Hazards ratios (HR) and 95% confidential interval (CI) were calculated. We selected variables in the best multivariate model with stepwise-selection (AIC minimum criteria).

The statistical analysis was performed with the R project software (21). The development and report of this predictive index was according to the TRIPOD (22) and STROBE (23) statements.

## Results

### Clinical Features of the Study Cohort

Eighty (66%) patients were men. The median age at recruitment was 48 (36–58) years. 34 (28%) patients were mild/moderate, 51 (42%) severe, and 36 (29%) critical. All patients completed the follow-up and were included in the analysis. 81 (66%) subjects had at least one comorbidity. 32 (26%) patients had diabetes, 31 (25%) hypertension, 48 (39%) obesity, 12 (9%) dyslipidemia, 8 (6%) cardiopathy, 4 (3%) chronic renal disease, 6 (4%) chronic liver disease, 4 (3%) chronic pulmonary disease, and 6 (4%) cancer. During the follow-up period, 22 (18%) patients died and 6 (5%) patients progressed. Patients with critical and severe COVID-19 had a higher body mass index (BMI) in comparison to mild disease (30.3 (27.45-34.12) *vs* 29.6 (27.35-31.68) *vs* 26.3 (24.9-29.1), *P=0.0008*, respectively). Conversely, mild/moderate patients had higher levels of hemoglobin (16.35 g/dL (16.35-16.35) *vs* 15.50 (14.2-16.35) *vs* 14.55 (11.55-16.12), *P<0.0001*) and albumin (4.55 g/dL (4.39-4.60) *vs* 3.80 (3.53-4.19) *vs* 3.12 (2.80-3.45), *P<0.0001*) in comparison to severe and critical subjects (**Table 1**).

**Table 1:** Clinical features of patients with COVID-19 according to disease severity.

Variable	Mild/moderate Median (IQR) N = 34	Severe Median (IQR) N = 51	Critical Median (IQR) N = 36	*P*-value
**Demographic features**				
Female (%)	44.2	39.3	16.7	
Male (%)	55.80	60.70	83.30	
Age (years)	34.00 (27.25-43.00)	48.00 (42.00-60.50)	54.50 (46.75-60.75)	*<0.001*
Comorbidities (Number)	0 (0-0)	1 (1-2)	1 (1-2)	*<0.001*
**Clinical features**				
Body mass index (kg/m^2^)	26.73 (24.90-29.10)	29.60 (27.35-32.68)	30.30 (27.45-34.12)	*<0.001*
Mean Arterial pressure (mmHg)	93.30 (85.25-100.00)	94.60 (84.80-101.50)	90.30 (82.72-100.00)	*0.60*
Heart rate (beats per minute)	92.00 (79.00-105.02)	105.00 (92.5-117.00)	113.5 (98.00-128.20)	*<0.001*
Respiratory rate (breaths per minute)	18.00 (16.00-20.00)	24.00 (20.00-30.00)	36.00 (30.00-42.00)	*<0.001*
Oxygen saturation (SpO2, %)	95.00 (95.00-94.00)	88.00 (84.00-90.50)	64.00 (53.50-82.25)	*<0.001*
Temperature (°C)	37.00 (36.50-37.50)	37.00 (36.50-37.50)	37.10 (36.2-37.52)	*0.59*
**Laboratory parameters**				
Leukocytes (cells/mm^3^)	7356.00 (5200.00-7912.00)	7300 (5250-10450)	10700.00 (7700.00-13075)	*<0.001*
Total lymphocytes (cells/mm^3^)	1478.88 (1157.25-1678.40)	837.00 (530.50-1143.50)	695.00 (503.00-977.00)	*<0.001*
Total neutrophils (cells/mm^3^)	3715.00 (3303-3715)	6042 (3919-8568)	9158.00 (6896.00-11390)	*<0.001*
Total monocytes (cells/mm^3^)	395.00 (395.00-480.80)	478 (303.50-644.50)	532.5 (377.20-663.50)	*0.14*
NT/LT ratio	2.50 (2.10-4.27)	8.50 (4.23-12.89)	12.27 (6.88-19.34)	*<0.001*
Hemoglobin (g/dL)	16.35 (16.35-16.35)	15.5 (14.20-16.35)	14.55 (11.55-16.12)	*<0.001*
Platelets (x10^3^ cells/mm^3^)	217.80 (217.80-217.80)	212.00 (176.00-262.50)	250.00 (198.80-345.00)	*0.032*
Glucose (mg/dL)	100.13 (96.18-106.54)	111.00 (101.5-125.50)	147.00 (113.20-199.50)	*<0.001*
Blood Urea Nitrogen (mg/dL)	12.09 (11.74-13.11)	13.30 (10.00-19.65)	21.90 (13.50-31.68)	*<0.001*
Creatinine (mg/dL)	0.88 (0.86-0.89)	0.94 (0.78-1.16)	0.95 (0.70-1.23)	*0.57*
Sodium (mmol/L)	137.80 (137.20-138.00)	136.00 (134.00-139.00)	136.00 (133.00-139.00)	*0.062*
Potassium (mmol/L)	4.06 (4.04-4.09)	4.02 (3.69-4.38)	4.11 (3.63-4.53)	*0.72*
Aspartate aminotransferase (U/L)	23.00 (18.10-32.00)	37.10 (26.95-64.35)	45.80 (32.27-63,70)	*<0.001*
Alanine aminotransferase (U/L)	31.45 (24.71-36.89)	38.3 (23.65-59.00)	38.40 (26.30-63.58)	*0.025*
Alkaline phosphatase (U/L)	80.43 (77.99-85.76)	85.00 (68.00-112.00)	95 (76.75-130.00)	*0.031*
Total bilirubin (mg/dL)	0.56 (0.33-0.62)	0.62 (0.50-0.79)	0.71 (0.42-1.03)	*0.055*
Direct bilirubin (mg/dL)	0.13 (0.08-0.15)	0.18 (0.13-0.21)	0.71 (0.42-1.03)	*0.001*
Indirect bilirubin (mg/dL)	0.42 (0.26-0.47)	0.45 (0.35-0.58)	0.46 (0.28-0.67)	*0.31*
Albumin (g/dL)	4.55 (4.39-4.60)	3.80 (3.53-4.19)	3.12 (2.80-3.45)	*<0.001*
Globulins (g/dL)	2.99 (2.96-3.04)	3.21 (2.88-3.53)	3.03 (2.75-3.27)	*0.058*
C reactive protein (mg/dL)	2.08 (0.96-4.21)	9.43 (6.04-14.24)	18.36 (8.93-26.72)	*<0.001*
Ferritin (ng/dL)	237.03 (200.20-334.80)	464.00 (241.50-757.00)	781.5 (431.80-1167.00)	*<0.001*
Troponin I (pg/mL)	2.80 (2.08-3.55)	5.10 (3.10-7.20)	14.80 (5.97-89.00)	*<0.001*
Lactate dehydrogenase (U/L)	195.5 (180.4-217.50)	344.00 (277.50-440.5)	518.5 (350.5-1115.00)	*<0.001*
Creatine phosphokinase (U/L)	89.56 (80.44-114.50)	141.00 (54.00-237.00)	155.00 (97.25-576.25)	*0.006*
D Dimer (ng/mL)	349.60 (312.90-417.80)	529.00 (417.00-933.50)	1375.00 (1070.00-2509.00)	*<0.001*
Thrombin time (Seconds)	10.40 (10.40-10.65)	12.50 (11.65-13.25)	12.25 (11.50-13.38)	*<0.001*
Thromboplastin time (Seconds)	29.9 (29.9-29.9)	32.70 (30.45-34.50)	32.75 (28.90-38.40)	*<0.001*
International Normalized Ratio	1.06 (1.06-1.06)	1.10 (1.00-1.14)	1.10 (1.00-1.20)	*0.019*
Fibrinogen (mg/dL)	370.20 (355.80421.00)	615.00 (460.50-723.00)	645.00 (540.00-808.00)	*<0.001*
**Arterial blood gases**				
pH	7.46 (7.46-7.46)	7.46 (7.44-7.48)	7.44 (7.38-7.47)	*0.0346*
PaCO2 (mmHg)	28.92 (28.92-28.92)	30.75 (28.35-32.20)	31.40 (28.93-39.10)	*0.001*
HCO3 (mmHg)	20.34 (20.34-20.34)	21.70 (20.00-22.70)	21.70 (18.95-25.62)	*0.006*
Lactate (mmol/L)	1.14 (1.14-1.14)	1.2 (1.00-1.60)	1.85 (1.47-2.65)	*<0.001*
PaFi	304.80 (304.80-304.80)	247.00 (221.00-296.50)	102.50 (86.50-166.80	*<0.001*
Anion Gap (mmol/L)	14.70 (14.70-14.70)	14.00 (12.90-15.45)	14.10 (12.10-16.25)	*0.13*

### Multiparametric Flow Cytometry Analysis of PBMCs Subsets

In **Table 2**, we depict the median (IQR) of every cell subset according to disease severity. Patients with critical COVID-19 had a lower absolute number of Th1 cells (19.18 (6.17-78.71) *vs* 100.05 (52.23-240.89), *P<0.0001*), whilst mild/moderate patients showed higher amounts of CD8^+^ effector memory T cells in comparison to severe and critical COVID-19 (34.45 (17.28-41.53) *vs* 22.29 (5.16-28.58) *vs* 1.25 (0.31-2.41), *P=0.0002*). In the myeloid compartment, total LDGs augmented according to disease severity (42.15 (22.01-94.11) *vs* 181 (65.7-319.40) *vs* (1062 (334.00-3427.20), *P<0.0001*) (**Table 2**).

**Table 2:** Peripheral blood cellular subsets of patients with COVID to 19 according to disease severity.

Variable	Mild/moderate Median (IQR) N = 34	Severe Median (IQR) N = 51	Critical Median (IQR) N = 36	*P*-value
**T and B lymphocyte subsets (absolute numbers)**				
Total CD4^+^ (cells/µl)	342.18 (163.47-534.17)	209.33 (119.67-354.47)	260.06 (113.36-344.01)	*0.090*
CD4^+^ regulatory cells (cells/µl)	105.41 (57.37-226.86)	89.82 (36.48-158.79)	100.59 (46.47-185.38)	*0.54*
Naïve CD4^+^ (cells/µl)	184.77 (98.78-322.36)	126.22 (64.15-211.21)	134.15 (72.34-241.80)	*0.13*
Total memory CD4^+^ (cells/µl)	78.88 (44.15-125.65)	49.79 (27.95-92.55)	54.52 (23.11-92.77)	*0.14*
Central memory CD4^+^ (cells/µl)	6.91 (3.25-12.29)	5.45 (1.20-10.14)	9.04 (1.61-19.85)	*0.43*
Effector memory CD4^+^ (cells/µl)	34.45 (17.28-56.64)	26.73 (11.47-35.90)	16.62 (5.01-40.89)	*0.0383*
Th1 (cells/µl)	100.05 (52.23-240.89)	50.14 (25.81-130.77)	19.18 (6.17-78.71)	*<0.001*
Th2 (cells/µl)	11.34 (7.04-28.51)	6.37 (3.65-15.86)	9.40 (4.16-21.01)	*0.0525*
Th17 (cells/µl)	2.65 (1.14-9.21)	1.52 (0.61-3.37)	0.52 (0.07-3.62)	*0.001*
Total CD8^+^ (cells/µl)	382.15 (212.51-484.74)	156.98 (80.94-258.45)	112.58 (71.25-183.34)	*<0.001*
CD8^+^ regulatory cells (cells/µl)	72.21 (35.61-113.28)	61.22 (28.67-130.60)	12.37 (6.20-27.83)	*<0.001*
Naïve CD8^+^ (cells/µl)	215.28 (131.01-302.85)	110.22 (50.02-184.44)	72.43 (40.38-121.03)	*<0.001*
Total memory CD8^+^ (cells/µl)	59.11 (31.64-118.06)	30.49 (14.26-44.92)	19.85 (6.11-43.16)	*<0.001*
Central memory CD8^+^ (cells/µl)	2.48 (1.25-5.48)	1.45 (0.55-2.98)	1.25 (0.31-2.41)	*0.090*
Effector memory CD8^+^ (cells/µl)	33.13 (17.05-77.15)	18.15 (5.16-28.58)	11.98 (1.12-24.49)	*<0.001*
Tc1 (cells/µl)	200.24 (64.35-311.67)	92.36 (35.09-137.55)	30.20 (5.73-71.50)	*<0.001*
Tc2 (cells/µl)	4.22 (2.48-11.73)	2.73 (1.13-5.52)	3.14 (1.81-9.61)	*0.065*
Tc17 (cells/µl)	2.64 (1.07-7.13)	0.60 (0.22-2.52)	0.29 (0.00-2.26)	*<0.001*
Total B cells (cells/µl)	30.83 (0.00-109.36)	67.15 (46.99-109.05)	69.73 (40.06-107.67)	*0.019*
Transitional CD21^-^ (cells/µl)	0.37 (0.00-2.55)	0.89 (0.31-1.74)	1.15 (0.28-3.46)	*0.15*
Transitional CD21^+^ (cells/µl)	0.29 (0.00-34.96)	1.23 (0.30-4.22)	0.41 (0.12-1.66)	*0.074*
Total naïve B cells	4.95 (0.00-21.21)	19.72 (13.56-30.66)	29.80 (10.67-44.22)	*0.003*
Resting naïve B cells	4.91 (0.00-20.89)	19.72 (13.36-30.46)	29.70 (10.57-43.99)	*0.002*
Activated naïve B cells	0.03 (0.00-0.25)	0.15 (0.06-0.32)	0.18 (0.05-0.40)	*0.001*
Total memory B cells	5.30 (0.00-15.78)	13.74 (6.53-20.61)	8.35 (4.94-12.65)	*0.007*
Unswitched classical memory (cells/µl)	0.44 (0.00-3.24)	1.56 (0.58-2.12)	1.22 (0.61-2.24)	*0.10*
Switched classical memory (cells/µl)	3.29 (0.00-13.53)	11.50 (5.59-16.27)	6.07 (2.78-9.86)	*0.002*
Plasmablasts (cells/µl)	0.43 (0.00-1.61)	1.28 (0.96-2.24)	0.92 (0.31-2.37)	*0.003*
Non-classic memory IgD^-^ (cells/µl)	1.00 (0.00-7.53)	5.35 (2.34-8.94)	3.36 (1.40-6.91)	*0.008*
Non-classic memory IgD^+^ (cells/µl)	0.48 (0.00-2.75)	2.23 (1.07-4.44)	1.99 (1.20-5.26)	*0.0236*
Mature B cells	10.55 (0.00-34.96)	33.62 (22.28-47.04)	41.17 (22.53-64.35)	*0.001*
Total Double Negative B cells	4.15 (0.00-13.37)	11.08 (5.62-15.74)	13.62 (6.64-20.00)	*<0.001*
Double Negative 1 (cells/µl)	0.23 (0.00-4.14)	3.56 (1.61-5.34)	0.85 (0.11-2.54)	*<0.001*
Double Negative 2 (cells/µl)	0.01 (0.00-0.20)	0.15 (0.03-0.88)	0.20 (0.05-1.08)	*<0.001*
Double Negative 3 (cells/µl)	1.73 (0.00-7.15)	6.05 (2.94-9.81)	9.36 (5.63-16.65)	*<0.001*
Double Negative 4 (cells/µl)	0.00 (0.00-0.00)	0.00 (0.00-0.01)	0.00 (0.00-0.04)	*0.10*
Total NK (cells/µl)	21.65 (13.50-33.08)	22.20 (15.70-29.80)	16.70 (8.99-31.02)	*0.26*
CD56^hi^ NK (cells/µl)	5.95 (3.16-9.78)	3.30 (2.00-5.48)	4.17 (1.68-7.18)	*0.060*
CD56^lo^ NK (cells/µl)	94.35 (89.22-96.75)	96.00 (94.60-98.00)	95.35 (92.83-98.35)	*0.066*
**Myeloid subsets**				
Classical Monocytes (cells/µl)	279.19 (249.84-351.35)	336.42 (211.19-481.48)	324.40 (244.30-467.50)	*0.33*
Intermediate Monocytes (cells/µl)	90.85 (51.84-118.01)	50.78 (25.89-104.63)	81.30 (31.19-167.20)	*0.12*
Non-classical Monocytes (cells/µl)	27.55 (23.46-42.15)	26.88 (12.64-54.46)	42.53 (14.41-74.71)	*0.69*
Total LDGs (cells/µl)	42.15 (22.01-94.11)	181.00 (65.70-304.90)	1062.90 (334.00-3427.20)	*<0.001*
CD10^+^ LDGs (cells/µl)	0.02 (0.00-0.10)	0.39 (0.07-4.10)	18.98 (1.45-295.87)	*<0.001*
CD10^-^ LDGs (cells/µl)	0.21 (0.05-0.85)	2.55 (0.66-6.68)	89.37 (8.95-206.53)	*<0.001*

### Serum Cytokine/Chemokine Profile

Other immunological variables, including serum cytokines/chemokines are summarized in **Table 3**. The serum levels of MCP-1 were higher in critical patients in comparison to mild/moderate subjects (592.23 (381.68-902.38) *vs* 473 (350-531.70), *P=0.013*).

**Table 3:** Serum Cytokines and chemokines from COVID-19 according to disease severity.

Variable	Mild/moderate Median (IQR) N = 34	Severe Median (IQR) N = 51	Critical Median (IQR) N = 36	*P*-value
IL-1a (pg/mL)	12.79 (7.16-55.52)	11.12 (0.75-22.31)	18.33 (8.36-24.56)	*0.11*
IL-1b (pg/mL)	2.66 (0.62-3.82)	1.14 (0.60-2.59)	3.24 (2.27-4.31)	*<0.001*
IL-1RA (pg/mL)	44.12 (32.49-80.61)	50.24 (27.68-106.72)	85.13 (43.79-154.59)	*0.018*
IL-2 (pg/mL)	1.68 (0.47-1.95)	0.80 (0.40-1.47)	1.86 (1.48-2.04)	*<0.001*
IL-3 (pg/mL)	0.69 (0.17-0.76)	0.19 (0.16-0.23)	0.72 (0.70-0.74)	*<0.001*
IL-4 (pg/mL)	58.73 (11.90-232.22)	11.90 (11.90-75.46)	50.96 (39.05-67.91)	*0.020*
IL-5 (pg/mL)	2.21 (0.57-4.41)	1.05 (0.65-2.16)	2.51 (1.94-4.55)	*0.001*
IL-6 (pg/mL)	12.44 (7.30-25.83)	20.42 (7.42-48.83)	41.07 (14.25-72.38)	*0.007*
IL-7 (pg/mL)	6.85 (2.50-12.18)	10.11 (5.24-16.75)	12.44 (9.42-17.70)	*0.0239*
IL-8 (pg/mL)	14.02 (8.23-19.44)	19.83 (11.71-45.45)	42.80 (30.59-174.19)	*<0.001*
IL-10 (pg/mL)	11.76 (9.77-17.80)	14.71 (8.58-22.23)	24.90 (11.69-34.74)	*0.045*
IL-12p70 (pg/mL)	4.63 (0.77-7.97)	1.33 (0.59-4.21)	4.49 (3.58-5.59)	*<0.001*
IL-12p40 (pg/mL)	12.40 (2.95-24.05)	2.95 (2.95-10.76)	14.87 (9.87-18.96)	*<0.001*
IL-13 (pg/mL)	3.42 (0.57-16.87)	0.56 (0.56-4.91)	3.42 (2.71-4.23)	*0.013*
IL-15 (pg/mL)	4.74 (3.33-6.35)	4.20 (2.26-6.37)	8.75 (6.79-10.36)	*<0.001*
IL-17A (pg/mL)	5.46 (2.36-7.40)	1.18 (0.60-5.46)	4.82 (3.30-6.52)	*0.002*
IL-18 (pg/mL)	516.7 (438.8-674.2)	555.9 (407.3-741.4)	934.00 (745.00-1127.30)	*<0.001*
TNF-a (pg/mL)	15.59 (10.66-20.54)	17.81 (15.22-25.43)	25.07 (17.82-35.41)	*<0.001*
TNF-b (pg/mL)	4.68 (0.70-25.20)	0.70 (0.70-10.56)	3.21 (2-61-3.86)	*0.11*
TGF- b1 (pg/mL)	82843.00 (69762.00-105076.00)	89804.00 (74158.00-100469.00)	88965.00 (74356.00-107545.00)	*0.67*
TGF-b2 (pg/mL)	3020.30 (932.9-3488.80)	932.88 (582.37-932.88)	3407.70 (2906.40-3515.80)	*<0.001*
TGF-b3 (pg/mL)	6753.22 (562.62-7444.03)	68.24 (68.24-68.24)	7444.03 (7444.03-7444.03)	*<0.001*
G-CSF (pg/mL)	37.28 (21.05-71.83)	10.10 (3.87-46.91)	35.08 (23.39-47.50)	*0.002*
GM-CSF (pg/mL)	5.49 (0.50-9.52)	0.50 (0.50-6.42)	7.07 (5.44-8.90)	*<0.001*
VEGF (pg/mL)	74.83 (34.5-104.38)	117.51 (57.83-175.21)	188.62 (93.28-293.08)	*<0.001*
EGF (pg/mL)	96.92 (60.84-181.22)	115.19 (74.25-178.27)	142.07 (76.38-216.85)	*0.43*
IFN-a2 (pg/mL)	31.60 (17.46-57.73)	10.90 (10.90-27.82)	34.59 (26.20-42.93)	*<0.001*
IFN-g (pg/mL)	17.91 (7.81-25.93)	6.64 (3.05-17-34)	8.30 (5.65-20.24)	*0.028*
MCP-1/CCL2 (pg/mL)	473.1 (350.40-531.70)	526.57 (382.62-683.87)	592.23 (381.68-902.38)	*0.017*
MIP-1a/CCL4 (pg/mL)	3.56 (0.70-7.91)	0.70 (0.70-7.76)	4.81 (3.41-8.08)	*0.016*
MIP-1b/CCL3 (pg/mL)	27.48 (16.84-34.37)	31.00 (22.36-42.28)	46.89 (27.36-66.78)	*<0.001*
IP-10/CXCL10 (pg/mL)	1002.90 (576.00-1769.30)	1766.40 (644.00-3625.90)	2826.6 (761.90-4573.30)	*0.007*
Eotaxin/CCL11 (pg/mL)	101.11 (75.29-113.91)	89.23 (64.31-116.25)	74.28 (59.56-88.84)	*0.074*

### Assessment of Circulating NETs, Muscle Atrophy, Hypoxia and Oxidative Stress Markers

Circulating NETs complexes were higher in severe and critical patients (0.83 (0.46-0.84) *vs* 1.3 (0.89-1.73) *vs* 1.11 (0.85-1.62), *P=0.0004*) in comparison to mild/moderate subjects as well (**Table 4**). Interestingly, the plasma levels of TRIM63 augmented according to the disease severity (**Table 4**).

**Table 4:** Other biological markers in patients with COVID-19 according to disease severity.

Variable	Mild/moderate Median (IQR) N = 34	Severe Median (IQR) N = 51	Critical Median (IQR) N = 36	*P*-value
**Coagulation pathway factors (AU)**				
Protein C	593.84 (447.79-749.56)	622.26 (518.46-762.62)	458.50 (284.82-612.76)	*0.033*
Protein S	492.35 (348.08-666.91)	567.36 (440.58-693.27)	436.56 (264.74-672.02)	*0.32*
Von Willebrand Factor	331.93 (290.52-443.79)	293.81 (211.85-388.86)	456.50 (246.86-649.54)	*0.015*
Factor IX	349.67 (234.93-515.41)	433.18 (289.10-534.95)	404.2 (262.50-502.60)	*0.56*
**Hypoxia markers**				
HIF-1a (pg/mL)	1742.50 (401.06-4394.40)	1022.00 (721.00-1862.00)	922.00 (501.70-1432.30)	*0.50*
8-hydroxy 2 deoxyguanosine (ng/mL)	30.77 (23.27-37.42)	42.55 (38.75-45.28)	31.15 (22.74-45.30)	*<0.001*
**Muscle atrophy markers**				
Atrogin-1 (ng/mL)	51.38 (26.68-77.31)	42.81 (27.29-56.28)	70.16 (36.89-93.03)	*0.017*
Trim63 (pg/mL)	103.6 (0.00-336.60)	266.01 (99.48-602.71)	918.60 (558.90-1096.80)	*<0.001*
**Neutrophil extracellular traps complexes (ODI)**				
NETs (DNA-NE)	0.83 (0.46-1.19)	1.39 (0.89-1.73)	1.11 (0.85-1.62)	*<0.001*

### Metabolomolic Signature

Metabolites that showed a fold change >0.5 (P adjusted <0.05) by clustering analysis according to disease severity are displayed in **Supplementary Figure 3** and **Supplementary Table 2**. Differences among groups are depicted in **Supplementary Table 3**. Interestingly, the serum levels of 3-Hydroxyisovaleric acid increased according to disease severity [1.78 (1.42-2.16) *vs* 2.54 (1.82-3.20) *vs* 3.86 (2.63-4.85), *P<0.0001*].

We developed enrichment and pathway analysis using the 15 metabolites identified in the clustering analysis. The main pathways altered were those related to the metabolism of glutamate/glutamine and alanine-aspartate-glutamate, Krebs cycle, and arginine biosynthesis (**Supplementary Figure 4**).

### Construction of the CLICOVID-19 SRS and the Adverse Outcomes Predictive Model

Variables included in the univariate analysis to construct the models for disease severity and prediction of adverse outcomes are depicted in **Supplementary Table 4**. The variables that individually predicted COVID-19 severity are shown in **Table 5**.

**Table 5:** Variables associated to COVID-19 severity included in the selected model.

Variable	β	S.E.	OR	95% CI	*P*-value
Body mass index	0.138	0.056	1.147	1.032-1.292	*0.014*
Hemoglobin g/dL	-0.835	0.144	0.433	0.259-0.651	*<0.001*
Albumin mg/dL	-2.105	0.429	0.121	0.031-0.374	*<0.001*
3 -hydroxyisovaleric (peak height)	0.623	0.230	1.864	1.248-3.137	*<0.001*
CD8+ effector memory T cells (cells/microliter)	-0.044	0.014	0.959	0.926-0.996	*0.001*
CD4+ Th1 (cells/microliter)	-0.011	0.004	0.988	0.980-0.996	*0.003*
Low- density granulocytes (cells/microliter)	0.002	0.001	1.002	1.001-1.004	*0.002*
Monocyte chemoattractant protein - 1 (pg/mL)	0.004	0.001	1.004	1.001-1.007	*<0.001*
TRIM63 (pg/mL)	0.003	0.001	1.002	1.0008-1.004	*0.004*
Neutrophil extracellular traps (ODI)	2.383	0.765	10.837	2.571-64.165	*0.001*

Missing data from hemoglobin and albumin from 19 patients were handled as described in Methods. There were not missing data from any of the other predictors.

The best supported model includes the BMI, hemoglobin, albumin, 3-Hydroxyisovaleric acid, absolute numbers of effector memory CD8+ T cells, Th1, LDGs and the serum/plasma concentrations of MCP-1, TRIM63, and NETs. The model was titled CLICOVID-19 SRS (CLinical and Immunological COVID-19 Severity Risk Score) and was defined as following:

$$\begin{array}{c} logit(Severity_{level})=0.138BMI-0.835Hb-2.105Alb+0.623\;OH\;isovaleric\\ -0.0044EMCD8-0.011Th1+0.002LDG+0.004MCP\\ +0.003Trim63+2.383NETs \end{array}$$

**Figure 1:**
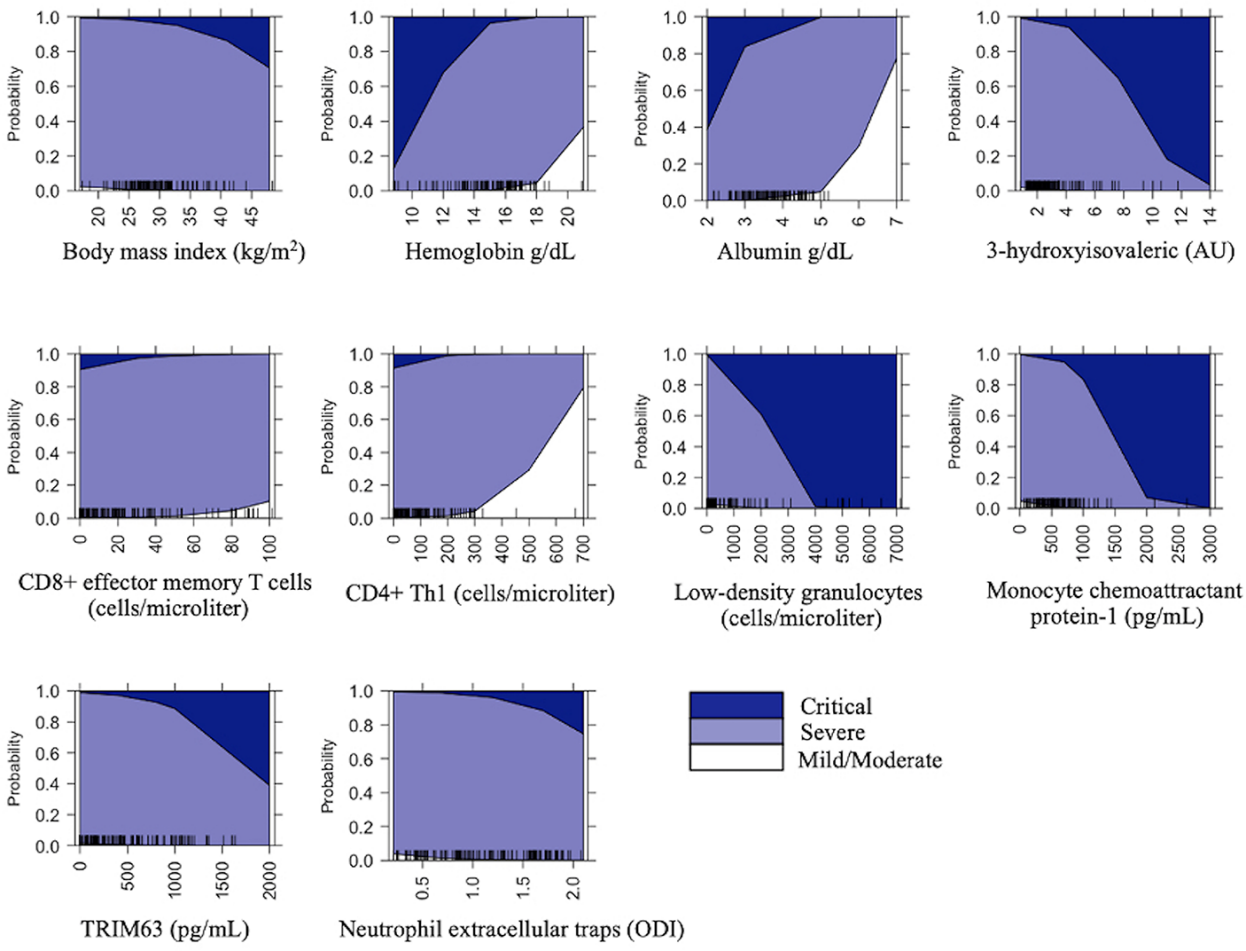
Prediction probabilities of COVID-19 severity for the variables included in the model. The graphs represent the size of the effect of each explanatory variable in the disease severity.

A graphical representation of the effect of the explanatory variables included in the model on disease severity is shown in **Figure 1**.

**Figure 2:**
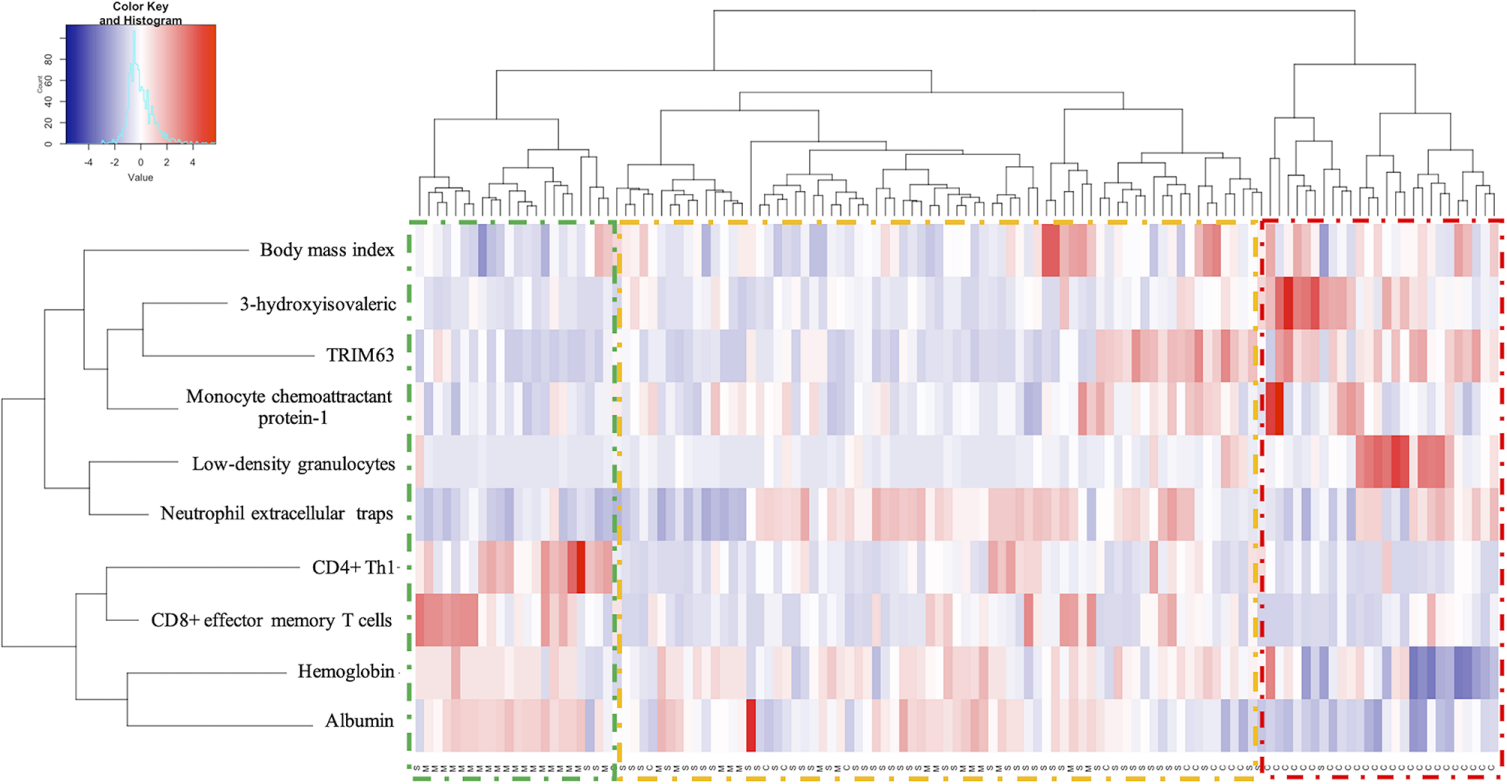
Heat map from clustering analysis for COVID-19 severity according-the predictive variables. Hierarchical clustering analysis generated by Ward´s method relating mild/moderate (M, green), severe (S, yellow) and critical (C, red).

The model predicts the probability for each level of disease severity depending on the values of the defining variables with an outstanding discriminative capacity with an optimism-corrected AUC (c-statistic) of 0.999 (**Supplementary Table 5**). As depicted in **Figure 2**, the index is able to separate the disease severity levels in clearly defined clusters.

The goodness of fit evaluated by the Lipsitz test was 5.12, *P=0.823*, which indicates low discrepancy between observed and expected values. Besides, the model had a good calibration as shown in **Supplementary Figure 6**. The model showed a sensitivity of 0.807 (0.547-1), specificity of 0.896 (0.773-1), PPV of 0.854 (0.627-1), NPV of 0.910 (0.793-1), +LR of 7.740 (2.408-7.882), and -LR of 0.216 (0.1-0.586).

The median (IQR) of follow-up time according to disease severity was: severe COVID-19 (73 (19.73) days), critical COVID-19 (52.5 (7-73) days). All mild/moderate were followed-up for 73 days. The variables that independently predicted adverse outcomes in COVID-19 patients are shown in **Table 6**.

**Table 6:** Multivariate Cox regression model to predict the risk of the development of adverse outcomes (progression and death) in patients with COVID-19.

Variable	Hazard ratio		95% Confidence interval	*P*
Severe COVID-19	4.18x10^4^	1.34x10^4^-1.31x10^5^		<0.0001
Critical COVID-19	5.35x10^5^	1.71x10^5^-1.67x10^6^		<0.0001
Body mass index	1.17	1.02-1.07		0.001
Neutrophil/lymphocyte ratio	1.05	1.02-1.07		<0.0001
Troponin I (pg/mL)	1.002	1.001-1.003		0.002
Lactate deshydrogenase (AU)	1.003	1.00-1.005		0.024
IP-10 (pg/mL)	1.00	1.00-1.001		0.001
3,4 dihydroxybutanoic acid (AU)	1.45	1.18-1.78		<0.0001
Malic acid (AU)	1.45	1.22-1.73		<0.0001
Citric acid (AU)	0.73	0.59-0.99		0.003
Myoinositol (AU)	0.97	0.95-0.99		0.008
Cystine (AU)	1.13	1.06-1.20		<0.0001

## Discussion

The main finding of the present work shows that a composite model which includes the BMI, hemoglobin, albumin, 3-Hydroxyisovaleric acid, absolute numbers of effector memory CD8^+^ T cells, Th1, LDGs and the serum/plasma concentrations of MCP-1, TRIM63, and NETs is a useful tool for the stratification of patients according to their disease severity. Our severity score has an outstanding discriminative capacity, showed good calibration, internal validity and unveils novel explanatory features for disease severity. Furthermore, our work unveils novel predictors of disease progression in COVID-19, including a distinctive metabolomic profile.

In COVID-19 obesity represents an independent risk factor for severe disease and admission to intensive care (24), which agrees with our study findings. This has been attributed to chronic inflammation mediated by adipose tissue and CD4^+^ lymphopenia with a biased phenotype towards Th17 and Th22 responses (24), which might explain the higher susceptibility for COVID-19 adverse outcomes.

According to our results, alterations in body composition are fundamental in the prognosis of COVID-19, since our study shows that muscle catabolism markers such as TRIM63 predict disease severity. ACE-2 is the receptor for SARS-CoV-2 and the activation of the renin angiotensin aldosterone system (RAS) favors muscle atrophy and fibrosis (25) through the transcription of the E3 ubiquitin ligase TRIM63 (25). Besides, it has been shown that critically ill patients with COVID-19 have necrosis, regeneration, atrophy and fatty infiltration of the muscle, which along with visceral adiposity, are risk factors for critical illness (26). To our knowledge, this is the first work that unveils the role of this ubiquitin ligase in COVID-19 severity.

The leucine catabolite 3-Hydroxyisovaleric acid has been found to be increased during hypoxic conditions (27). Agreeing with our results, raised serum levels of 3-Hydroxyisovaleric acid have been described in patients with severe COVID-19 and impaired lung function (27). This increase has been suggested to be secondary to an altered mitochondrial branched-chain amino acid (BCAA) metabolism related to hypoxia (27) and to the activation of the gluconeogenesis pathway. Indeed, COVID-19 severity and enhanced muscle catabolism have been associated with enhanced gluconeogenesis (27), which further supports our findings.

The cytokine storm is another acknowledged mechanism of tissue damage in COVID-19 and is driven by diverse cytokines and chemokines including MCP-1 and IP-10 (8), that are key mediators of lung damage through the chemotaxis of myeloid cells to the target tissues in viral infections (28). According to our results, IP-10 has been described as a key cytokine related to COVID-19 progression and its monitoring has been proposed as a promising tool for therapeutic decisions (29). MCP-1 is a chemokine involved in this cytokine storm and has been found to be elevated in sera of COVID-19 patients with respiratory failure (30). MCP-1 correlates with D-dimer and induces the synthesis of tissue factor which promotes thrombosis (31). Additionally, MCP-1 favors a Th2 phenotype and blocks the production of IFN-γ secreting cells (31), which agrees with our findings of diminished numbers of Th1 cells in severe COVID-19. In this regard CD8^+^ and CD4^+^ T cells from severe COVID-19 patients are known to have a lower cytotoxic activity and decreased production of IFN-γ respectively, which correlates with age and inflammatory parameters (32). Regarding this cytotoxic response, previous studies have shown that COVID-19 patients have decreased levels of CD8^+^ effector memory T cells (CD27-, CD45RA-) (33), which is corroborated by our findings. In animal models of lung viral infections, it has been shown that effector memory CD8^+^ T cells are key to an effective initial and subsequent immune response, which indicates that an adequate number of effector memory CD8^+^ T cells is essential in the anti-SARS-CoV-2 response. In fact, this cell subset is enriched in mild convalescent patients (34).

An abnormal antiviral T cell response allows the persistence of SARS-CoV-2 with the consequent over-activation of the innate immune response (35). In patients with severe COVID-19, a profound dysregulation of the myeloid compartment and emergency myelopoiesis have been demonstrated (36). Neutrophils are the key mediators of tissue damage in lung viral infections. In agreement with our results, previous studies have shown an increase in a cell subset similar to low-density granulocytes in COVID-19, particularly in critically ill subjects (37), which could be explained by the emergency myelopoiesis. According to our findings, NETs have been described as a key pathogenic factor in severe and critical COVID-19 (38).

Lower levels of hemoglobin and albumin are surrogate markers for the hyper-inflammatory state that drives COVID-19 severity. Anemia is found in approximately 31.3% of patients and is much more frequent in critical COVID-19 (39). Hypoalbuminemia (<3.5 g/dL) is detected in 74% of patients with severe COVID-19 and correlates with thrombotic complications (40). Both parameters were confirmed to be risk factors for COVID-19 severity in our study. Regarding disease progression, our study highlights the importance of LDH, troponin I (41) and neutrophil to lymphocyte ratio (42) as markers of severe inflammation and hypoxia-induced tissue damage and confirms their role as risk factors for adverse outcomes in COVID-19.

Our work confirms the role of increased levels of cystine, 3,4 dihydroxibutanoic and depletion of citric acid as independent risk factors for adverse outcomes in COVID-19 patients (43). On the other hand, malate levels increment could be related to the malate shuttle and malate dehydrogenase activities as a way to contend with increased NADH levels due to electron transport chain inhibition and increased glycolysis (44). Malate levels increment could be related to the malate shuttle and malate dehydrogenase activities as a way to contend with increased NADH levels due to electron transport chain inhibition and increased glycolisis (44). To our knowledge this is the first study to unveil the role of lower levels of myo inositol as a marker of disease progression in COVID-19. Depletion of myo inositol has been related to adverse outcomes in neonatal ARDS (45) and its supplementation has been associated with lower ROS levels in animal models (46), outcomes suggesting the potential beneficial effect of its supplementation.

Most of the COVID-19 severity explanatory models are not optimal since they are based exclusively on clinical variables assessed solely in hospitalized patients, with retrospective designs and without the inclusion of Latin American population. This is of great relevance, since the American continent is currently the epicenter of this pandemic and Mexico is one of the most affected countries in terms of confirmed cases, but mainly in the high lethality rate, which has been suggested to be related to the increased prevalence of metabolic comorbidities (47). Although this study is one of the few to include Latin-American patients, we acknowledge that our results should be confirmed in independent cohorts in order to be representative of this population. Our explanatory model shows a good calibration, internal validity and the highest discriminatory capacity in an indirect comparison with most of the published severity indexes (48–50). It also showed a well goodness of fit with lower discrepancy between observed and expected values, which highlights its utility.

To our knowledge, this is the first model that includes clinical features along with biological variables that represent the wide spectrum of the pivotal pathogenic phenomena related to disease severity in COVID-19. Under the current digital evolution driven by this pandemic, we decided to construct an online risk digital calculator based on the predictive index, which is publicly available (https://rai-unam-mx.shinyapps.io/CLICOVID-19-SRS/).

Our study has several limitations, including the sample size related to the high number of biological variables that were assessed, which was a barrier to include a larger amount of patients in whom all these variables could be measured. Nonetheless, the sample size allowed us to find differences among the severity groups. Additionally, this was a unicentric study, and the features included in our predictive index might only be available in tertiary care centers with access to flow cytometry and gas chromatography coupled to mass spectrometry (GC/MS). Another limitation of the study is the lack of information regarding the HLA genetic background of the patients and the SARS-CoV-2 viral load or genotype, which has been suggested to be contributors to COVID-19 severity (51). Our findings must be confirmed by the external validation in an independent cohort and in different settings. Moreover, to enhance the application of this predictive index, we are also developing a rapid reactive test to address the biological variables included in the index from a whole blood sample.

**Figure 3:**
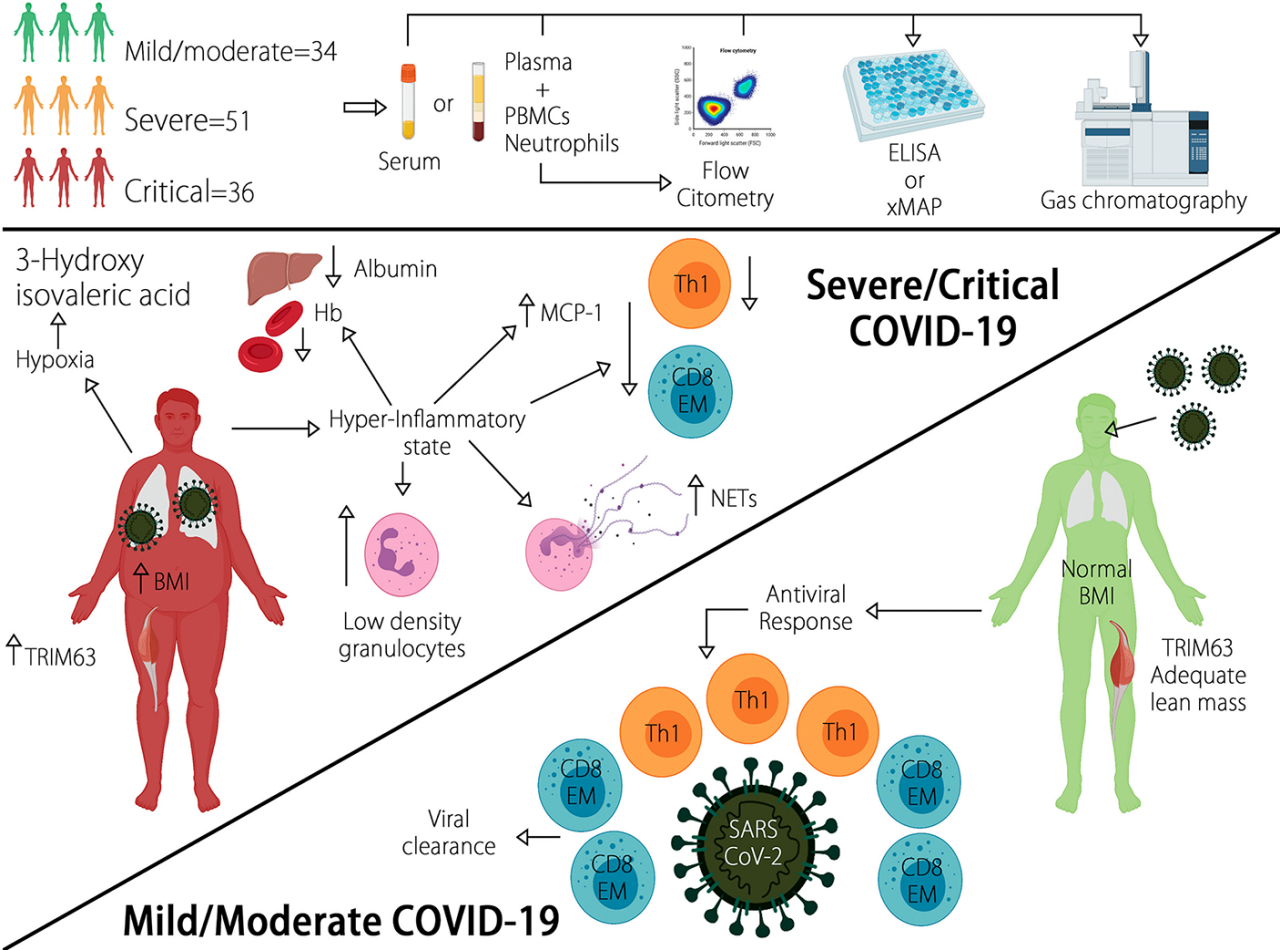
Diverse clinical and biological traits are able to accurately predict COVID-19 severity. Severe/critical disease is characterized by increased BMI, LDGs, circulating NETs, plasma TRIM63, and serum 3-Hydroxyisovaleric acid, as well as diminished levels of Hb, albumin, and lymphopenia of Th1 and CD8^+^ effector memory T cells.

In summary, the CLICOVID-19 SRS showed the best performance to accurately stratify the COVID-19 severity in both ambulatory and hospitalized patients in an indirect comparison with other predictive indexes. A representation of the results of this study in **Figure 3**. After external validation in different settings, the CLICOVID-19 SRS could be a useful tool to optimize the healthcare resources allocated to manage COVID-19 by identifying subjects that could be safely managed as outpatients and those in whom hospital admission and intensive care is imperative.

## Data Availability Statement 

The original contributions presented in the study are included in the article/**Supplementary Material**. Further inquiries can be directed to the corresponding author.

## Ethics Statement 

The studies involving human participants were reviewed and approved by Research and Ethics committees. The patients/participants provided their written informed consent to participate in this study.

## Author Contributions

JT-R, DG-M, and AP-F participated in the planning, conducting and reporting of the work. JM-M, NM-D, JP-F, SR-R, VS-H, AA-A, MN-A, GJ-V, and TH-G participated in the conducting of the work. AK-B participated in the creation of the on-line calculator and related app. AP-L and LL participated in the planning and reporting of the work. DG-M is the guarantor and responsible for the overall content of the paper. All authors contributed to the article and approved the submitted version.

## Funding

This study was funded by a research grant provided by Consejo Nacional de Ciencia y Tecnología (CONACYT, grant number 313252, F0005-2020-01), as well as a research grant from the Coordinación de Investigación Científica-UNAM, granted to JM-M. This grant covered the costs for the acquisition of reagents and appropriate equipment for some of the experimental procedures.

## Conflict of Interest

The authors declare that the research was conducted in the absence of any commercial or financial relationships that could be construed as a potential conflict of interest.

## Publisher’s Note

All claims expressed in this article are solely those of the authors and do not necessarily represent those of their affiliated organizations, or those of the publisher, the editors and the reviewers. Any product that may be evaluated in this article, or claim that may be made by its manufacturer, is not guaranteed or endorsed by the publisher.
