# Supplementary material for: Redefining COVID-19 Severity and Prognosis: The Role of Clinical and Immunobiotypes
Source: Front Immunol. 2021 Sep 8;12:689966. doi: 10.3389/fimmu.2021.689966 (PMC8456081; doi:10.3389/fimmu.2021.689966)
Supplement: Supplementary file 1 [file DataSheet_1.docx]

**Supplementary material**

*
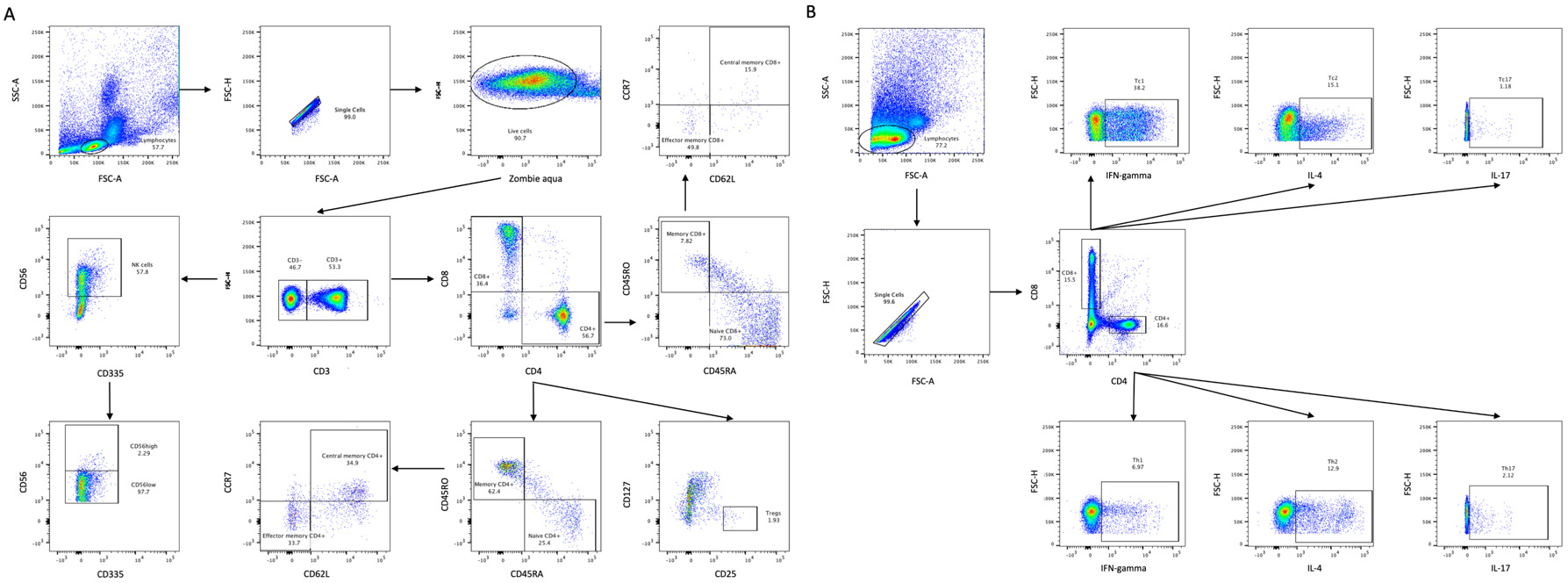
* **Supplementary figure 1.** Gating strategy for the evaluation of T cell subsets.

**
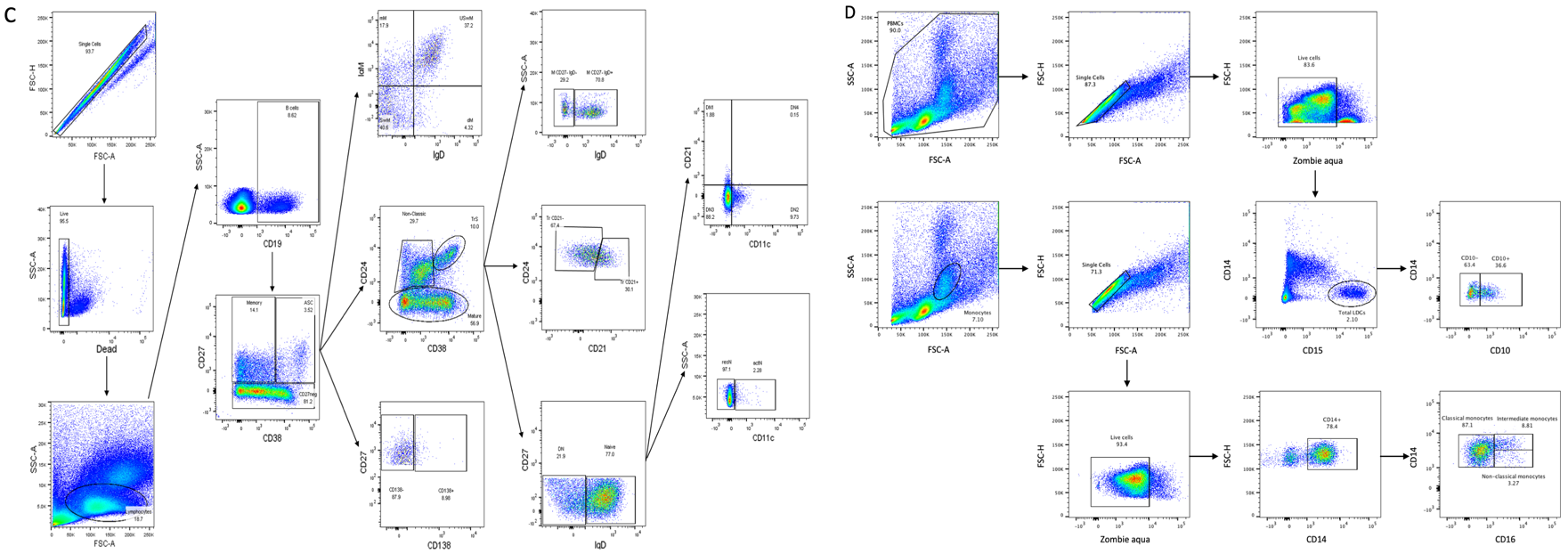
Supplementary figure 2.** Gating strategy for the evaluation of B and myeloid cell subsets

**Supplementary methods**

*Appraisal of the metabolomic signature*

We performed untargeted metabolomics analysis of sera from all patients employing gas chromatography coupled to mass spectrometry (GC/MS). Our metabolomic method detected 46 metabolites with relative standard deviation (RSD) <30% in the quality control (QC) sample, that consists of equal volumes of all the samples included in the analysis, as previously described^1^.

Thirty-five µL of serum were added in 150 µL of 1:3 chloroform-methanol. 5 µL of internal standard (IS - tetradecanoic acid, methyl tricosanoate, 5α-cholestane - 0.15 mg/mL) were added for quality control. Once mixed by vortex, the microtubes were incubated at -20°C for 20 min and centrifuged at 14000 rpm for 10 minutes. 150 µL of recovered supernatants were dried with nitrogen and the precipitate redissolved in 20 µL of methoxyamine dissolved in pyridine (20 mg/mL). Afterwards, the sample was incubated for 90 min at 37°C. After the incubation period, 40 µL of MBSTFA + 1% TMCS and a FAME mixture were added and incubated for 60 min at 37°C. One microliter was injected by splitless technique to a GC/MS (Agilent 5977A/7890B/G4513A, California, USA) and was run under the following settings: column flow 1ml/min, inlet temperature 200°C, source temperature 200°C, interface temperature 250°C. An Agilent HP5ms column (30 m x 250 µm x 0.25 µm) was employed with helium 99.9999 % as a mobile phase. The chromatographic method consisted of 1 min hold at 60°C with a ramp of 10°C/min until 325°C, with a final held time of 10 min.

The IS was used to identify variations in retention times and sensitivity during the analysis. To evaluate the reproducibility of the analytical method, we employed a Quality Control sample (generated by mixing equal volumes of all the samples included in the study) that was injected every five samples per batch and day of analysis. Only the metabolites with RSD lower than 30% were selected as previously described^1^. The average RSD value for all the metabolites included in the analysis was 13.9%.

**Deconvolution and identification**

Once acquired, the raw data was transformed to mzdata using Chemstation (Agilent). Mzmine2 software was employed to perform the deconvolution process using the next parameters: RT range: 6.5-29.5 min; m/z range: 50-500; noise level: 1x10^3^; m/z tolerance: 0.5; and peak range: 0.01-0.2 min. The data was filtered according to the rule of 80%.

The metabolite identifications were obtained with the NIST 2.0 library. Only results with R>700 were considered for further analysis.

**Supplementary table 1. List of PBMCs subsets and their respective markers.**

| **Cells subsets** | **Cells markers** |
| --- | --- |
| CD4^+^ T cells | CD3^+^, CD4^+^ |
| CD8^+^ T cells | CD3^+^, CD8^+^ |
| Naïve T cells CD4^+^ | CD3^+^, CD4^+^, CD45RA^+^, CD45RO^-^ |
| Naïve T cells CD8^+^ | CD3^+^, CD8^+^, CD45RA^+^, CD45RO^-^ |
| Memory T cells CD4^+^ | CD3^+^, CD4^+^ ,CD45RA^-^, CD45RO^+^ |
| Memory T cells CD8^+^ | CD3^+^, CD8^+^ CD45RA^-^, CD45RO^+^ |
| Central memory T cells CD4^+^ | CD3^+^. CD4^+^ ,CD45RA^-^, CD45RO^+^, CD62L^+^, CCR7^+^ |
| Central memory T cells CD8^+^ | CD3^+^, CD8^+^ ,CD45RA^-^, CD45RO^+^, CD62L^+^, CCR7^+^ |
| Effector memory T cells CD4^+^ | CD3^+^, CD4^+^, CD45RA^-^, CD45RO^+^, CD62L^-^, CCR7^-^ |
| Effector memory T cells CD8^+^ | CD3^+^, CD8^+^, CD45RA^-^, CD45RO^+^, CD62L^-^, CCR7^-^ |
| T helper 1 | CD3^+^, CD4^+^, IFN-γ^+^ |
| T helper 2 | CD3^+^, CD4^+^, IL-4^+^ |
| T helper 17 | CD3^+^, CD4^+^, IL-17^+^ |
| T cytotoxic 1 | CD8^+^, IFN-γ^+^ |
| T cytotoxic 2 | CD8^+^, IL-4^+^ |
| T cytotoxic17 | CD8^+^, IL-17^+^ |
| NK cells | CD3^-^, CD335^+^, CD56^hi/lo^ |
| NK cells with high expression of CD56 | CD3^-^, CD335^+^**,**CD56^hi^ |
| NK cells with low expression of CD56 | CD3^-^, CD335^+^,CD56^lo^ |
| Regulatory T cells | CD4^+^, CD25^hi^, CD127^lo/-^ |
| Total B cells | CD3^-^, CD19^+^ |
| Transitional B cells CD21^-^ | CD19^+^, CD27^-^, CD38^hi^, CD24^hi^, CD21^-/lo^ |
| Transitional B cells CD21^+^ | CD19^+^, CD27^-^, CD38^hi^, CD24^lo^, CD21^+^ |
| Total naïve B cells | CD19^+^, CD27^-^, IgD^+^, CD38^-^, CD24^-^, CD11c^-/+^ |
| Resting naïve B cells | CD19^+^, CD27^-^, IgD^+^, CD38^-^, CD24^-^, CD11c^-^ |
| Activated naïve B cells | CD19^+^, CD27^-^, IgD^+^, CD38^-^, CD24^-^, CD11c^+^ |
| Plasmablasts | CD19^+^, CD27^hi^, CD38^hi^ |
| Total memory B cells | CD19^+^, CD27^+^, IgD^+/-^ |
| Unswitched classical memory B cells | CD19^+^, CD27^+^, IgD^+^ |
| Switched classical memory B cells | CD19^+^, CD27^+^, IgD^-^ |
| Non-classical CD27^-^ IgD^-^ memory B cells | CD19^+^, CD38^-/lo^, CD24^+^, CD27^-^, IgD^-^ |
| Non-classical CD27^-^ IgD^+^ memory B cells | CD19^+^, CD38^-/lo^, CD24^+^, CD27^-^, IgD^+^ |
| Total double negative B cells | CD19^+^, CD27^-^, IgD^-^, CD38^-^, CD24^-^, CD21^+/-^, CD11c^-/+^ |
| Double negative 1 B cells | CD19^+^, CD27^-^, IgD^-^, CD38^-^, CD24^-^, CD21^+^, CD11c^-^ |
| Double negative 2 B cells | CD19^+^, CD27^-^, IgD^-^, CD38^-^, CD24^-^, CD21^-^, CD11c^+^ |
| Double negative 3 B cells | CD19^+^, CD27^-^, IgD^-^, CD38^-^, CD24^-^, CD21^-^, CD11c^-^ |
| Double negative 4 B cells | CD19^+^, CD27^-^, IgD^-^, CD38^-^, CD24^-^, CD21^+^, CD11c^+^ |

**Supplementary table 2. List of metabolites with Fold Change >0.5 among the COVID-19 severity groups.** Fold changes values for Mild/moderate vs Severe, Mild/moderate vs Critical and Severe vs Critical COVID-19 patients (P adjusted <0.05). The analysis was performed with Metaboanalyst 5.0.

| **Severity** | **Metabolite** | **HMDB** | **FC** | **log2(FC)** | **Adjusted P** |
| --- | --- | --- | --- | --- | --- |
| **Mild/Moderate vs Severe** | α-Hydroxyisovaleric acid | HMDB0000407 | 1.8766 | 0.90811 | 0.00010265 |
|  | 3-Hydroxybutyric acid | HMDB0000011 | 1.7969 | 0.84553 | 0.0063978 |
|  | α-Hydroxybutyric acid | HMDB0000008 | 1.6611 | 0.73215 | 0.00010265 |
| **Mild/moderate vs Critical** | 3-Hydroxyisovaleric acid | HMDB0000754 | 2.5292 | 1.3387 | 5.59E-08 |
|  | α-Hydroxyisovaleric acid | HMDB0000407 | 2.1802 | 1.1245 | 2.42E-05 |
|  | Pseudouridine | HMDB0000767 | 1.9267 | 0.9461 | 0.0024018 |
|  | Glycerol | HMDB00131 | 1.8128 | 0.85824 | 0.0083319 |
|  | 2,3-Dihydroxybutanoic acid | HMDB0002453 | 1.807 | 0.85357 | 0.030411 |
|  | α-Ketoglutarate | HMDB0000208 | 1.6067 | 0.68408 | 0.0048914 |
|  | α-Hydroxybutyric acid | HMDB0000008 | 1.6004 | 0.67845 | 3.70E-05 |
|  | Glutamine | HMDB0000641 | 0.60511 | -0.72472 | 8.61E-08 |
|  | 1,5-anhydro-D-sorbitol | HMDB0002712 | 0.57867 | -0.78919 | 5.97E-05 |
| **Severe vs Critical** | 3-Hydroxyisovaleric acid | HMDB0000754 | 1.7705 | 0.82419 | 0.0015666 |
|  | α-Ketoglutarate | HMDB0000208 | 1.6461 | 0.71902 | 0.0010073 |
|  | Glycerol | HMDB0000131 | 1.6296 | 0.70452 | 0.018344 |
|  | Glutamic acid | HMDB0000148 | 1.5913 | 0.67022 | 0.00035785 |
|  | Pseudouridine | HMDB0000767 | 1.5625 | 0.64382 | 0.07681 |
|  | Glutamine | HMDB0000641 | 0.6663 | -0.58576 | 0.00010385 |
|  | Cystine | HMDB0000192 | 0.50106 | -0.99695 | 1.8099E-05 |

**Supplementary table 3.** Metabolomic profile of patients with COVID-19 according to disease severity.

| **Variable** | **Mild/moderate**  **Median (IQR)**  **N=34** | **Severe**  **Median (IQR)**  **N=51** | **Critical**  **Median (IQR)**  **N=36** | ***P*-value** |
| --- | --- | --- | --- | --- |
| Pyruvate | 2.30 (0.79-3.03) | 1.61 (0.93-2.64) | 2.73 (1.05-3.66) | *0.25* |
| Glycolic acid | 0.39 (0.35-0.46) | 0.37 (0.33-0.46) | 0.39 (0.32-0.49) | *0.67* |
| 2 - keto -3-methylvaleric acid | 371 (3.36-4.27) | 4.22 (3.60-4.68) | 2.87 (2.52-3.69) | *<0.001* |
| α - Hydroxybutyric acid | 30.11 (24.28-43.49) | 53.53 (42.12-68.83) | 51.34 (40.24-66.14) | *<0.001* |
| 3 - hydroxybutyric acid | 9.74 (5.82-17.17) | 19.45 (10.12-33.23) | 10.21 (5.18-20.37) | *0.006* |
| α - Hydroxyisovaleric acid | 4.86 (3.97-6.63) | 8.30 (5.73-11.75) | 8.80 (6.50-15.43) | *<0.001* |
| b -alanine | 1.84 (1.24-2.60) | 1.52 (1.27-2.05) | 1.96 (1.34-2.72) | *0.14* |
| 3 - Hydroxyisovaleric acid | 1.78 (1.42-2.16) | 2.54 (1.82-3.20) | 3.86 (2.63-6.35) | *<0.001* |
| Valine | 32.84 (28.79-36.05) | 34.01 (31.60-36.37) | 31.66 (26.88-35.22) | *0.028* |
| Leucine | 17.84 (14.69-19.09) | 19.53 (18.13-20.98) | 16.98 (13.76-18.70) | *<0.001* |
| Glycerol | 16.16 (13.13-20.15) | 16.64 (13.23-21.20) | 21.74 (16.66-39.25) | *0.004* |
| Isoleucine | 52.89 (41.94-63.56) | 59.71 (52.09-67.05) | 55.21 (47.73-62.95) | *0.11* |
| Proline | 20.65 (18.13-26.34) | 17.57 (14.68-20.50) | 15.82 (14.12-18.83) | *<0.001* |
| Pipecolinic acid | 1.88 (1.54-2.29) | 1.88 (1.41-2.53) | 1.42 (1.16-1.98) | *0.006* |
| Glyceric acid | 0.11 (0.10-0.15) | 0.08 (0.06-0.10) | 0.13 (0.10-0.16) | *<0.001* |
| 2,3 - dihydroxybutanoic acid | 1.16 (0.81-2.10) | 1.82 (1.16-2.48) | 2.01 (1.38-3.17) | *0.014* |
| Serine | 71.42 (66.85-76.06) | 72.03 (61.21-77.48) | 68.62 (57.65-74.11) | *0.26* |
| Threonine | 50.33 (45.55-57.10) | 50.42 (42.74-61.09) | 47.55 (47.28-54.97) | *0.074* |
| 3,4 -dihydroxybutanoic acid | 2.37 (1.95-2.91) | 2.20 (1.84-2.72) | 2.66 (1.99-4.26) | *0.22* |
| Malic acid | 3.37 (2.70-4.36) | 3.01 (2.36-3.88) | 4.29 (3.22-5.15) | *0.001* |
| Methionine | 2.20 (1.80-2.44) | 2.25 (1.91-2.59) | 2.25 (1.94-2.71) | *0.32* |
| 5 - oxoproline | 62.12 (57.90-68.50) | 57.02 (53.02-63.87) | 57.62 (52.11-62.47) | *0.021* |
| Cysteine | 7.63 (5.66-8.72) | 6.49 (5.29-8.10) | 5.21 (3.97-8.98) | *0.070* |
| Threonic acid | 8.00 (4.32-10.91) | 6.85 (5.81-9.26) | 4.63 (3.62-6.57) | *0.002* |
| a-ketoglutarate | 4.50 (3.79-6.36) | 4.55 (3.70-5.88) | 6.56 (4.81-9.72) | *<0.001* |
| Ornithine | 3.73 (2.56-4.70) | 3.66 (2.73-4.50) | 3.33 (2.35-4.39) | *0.46* |
| Glutamic acid | 49.47 (38.57-63.64) | 41.16 (32.24-59-15) | 73.51 (55.09-99.43) | *<0.001* |
| Phenylalanine | 40.87 (35.13-45.95) | 41.81 (38.58-50.10) | 48.97 (44.96-53.71) | *<0.001* |
| Lysine | 23.39 (15.01-33.94) | 24.57 (17.94-34.76) | 20.43 (11.36-30.36) | *0.063* |
| Glutamine | 70.00 (60.53-83.26) | 67.28 (54.13-80.59) | 44.36 (33.07-57.57) | *<0.001* |
| Azelaic acid | 10.26 (7.44-16.24) | 7.24 (5.08-10.71) | 10.70 (7.97-15.25) | *0.003* |
| Hypoxanthine | 4.15 (3.29-5.40) | 4.32 (3.61-5.19) | 4.41 (3.03-7.79) | *0.79* |
| Ornithine | 18.95 (12.93-30.52) | 18.29 (11.86-23.60) | 20.65 (16.61-26.94) | *0.087* |
| Citric acid | 6.56 (5.69-8.15) | 4.92 (4.15-6.89) | 4.20 (2.78-6.10) | *<0.001* |
| Myristic acid | 3.19 (2.53-4.05) | 3.25 (2.55-4.07) | 2.97 (2.35-3.91) | *0.61* |
| 1,5 -anhydro to D – sorbitol | 78.37 (57.25-103.17) | 58.02 (36.16-69.70) | 47.36 (34.5-57.63) | *<0.001* |
| Tyrosine | 60.12 (54.71-66.77) | 54.41 (46.61-63.01) | 63.00 (49.49-74.26) | *0.056* |
| Palmitic acid | 69.92 (52.31-82.36) | 71.80 (62.92-80.42) | 70.74 (63.10-79.07) | *0.39* |
| Myo-inositol | 13.94 (11.33-18.28) | 15.54 (12.78-20.16) | 14.55 (9.50-40.54) | *0.39* |
| Heptadecanoic acid | 1.01 (0.85-1.13) | 0.99 (0.80-1.15) | 1.04 (0.82-1.41) | *0.63* |
| Oleic acid | 4.22 (2.62-6.32) | 5.98 (4.24-6.96) | 4.36 (3.52-6.23) | *0.001* |
| Stearic acid | 51.17 (45.93-56.51) | 47.23 (44.71-55.23) | 49.23 (42.09-54.07) | *0.43* |
| Cystine | 12.64 (5.55-14.94) | 16.46 (12.92-19.52) | 5.25 (3.50-12.61) | *<0.001* |
| Pseudouridine | 1.58 (1.46-2.06) | 1.87 (1.60-2.33) | 2.32 (1.84-3.19) | *0.001* |
| b-tocopherol | 4.05 (3.20-5.50) | 3.74 (3.06-5.12) | 3.53 (2.82-4.83) | *0.43* |
| Cholesterol | 26.03 (23.42-28.15) | 22.01 (20.25-24.75) | 21.98 (19.17-24.40) | *<0.001* |

**Supplementary figure 3. Changes in metabolites according to disease severity.** Volcano plots based on Fold Change and P values of metabolites that increase (red) or decrease (blue) in a) mild/moderate vs severe COVID-19, b) mild/moderate vs critical COVID-19 and c) severe vs critical COVID-19. Cut off fold change > 0.5, p-value <0.05. d) Heatmap and hierarchical clustering analysis (p values - ANOVA) of differential metabolites among critical (C), mild /moderate (M) and severe (S) COVID-19 patients.


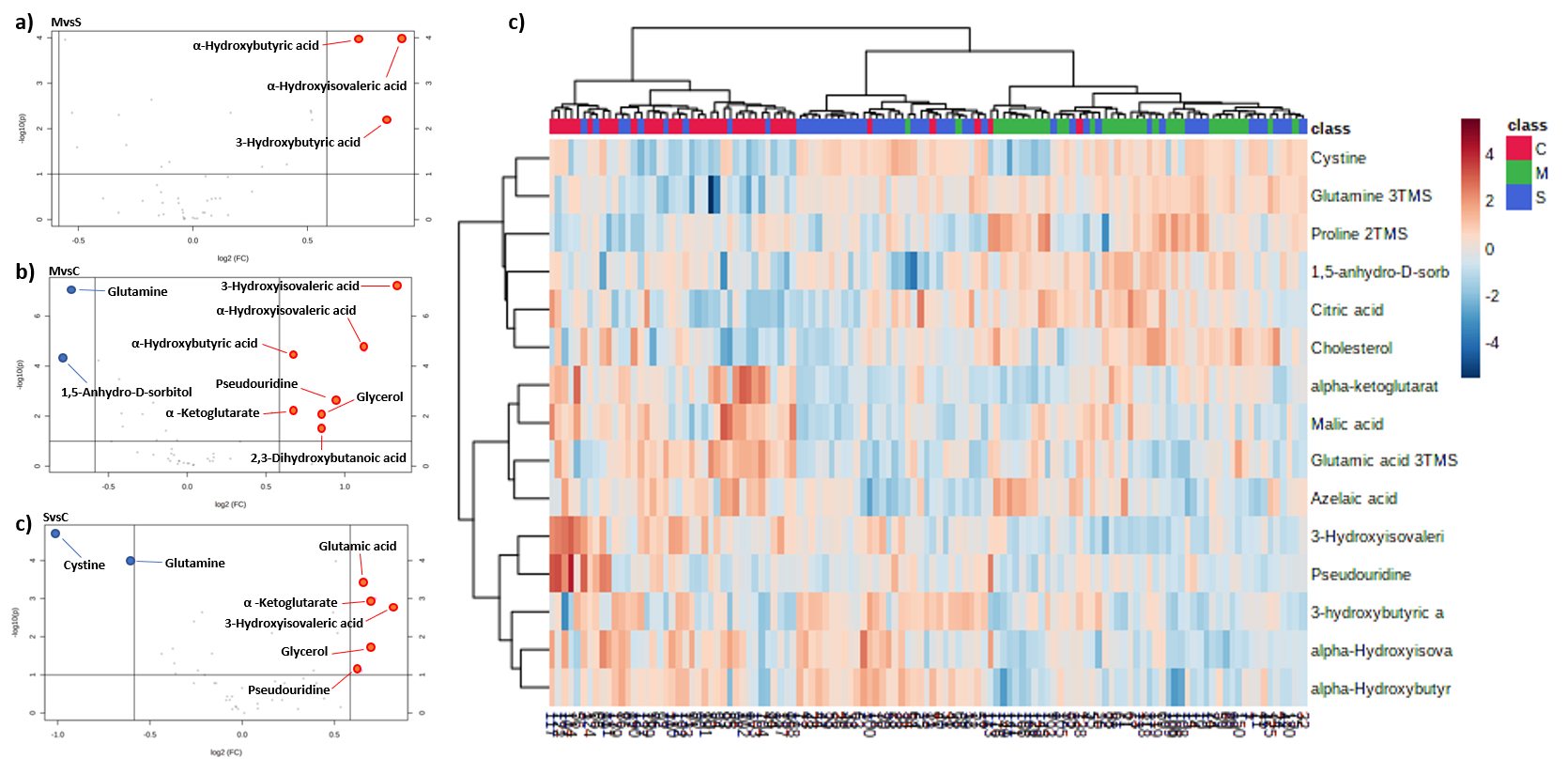


**Supplementary figure 4. Pathway mapping based on metabolites from hierarchical clustering and heatmap analysis.** Enrichment (a) and metabolic pathway analysis (b) from serum metabolomic profile of mild/moderate, severe and critical patients. Only the pathways with p<0.05 are shown.

**
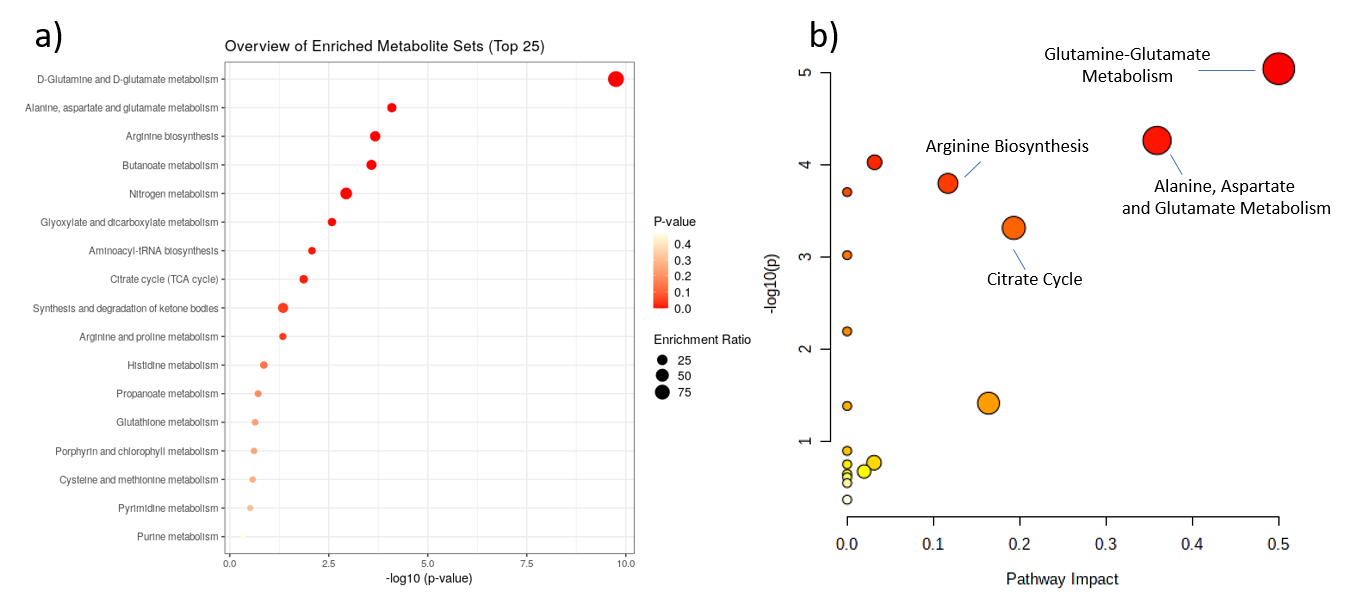
**

Metaboanalyst 5.0 was employed to generate the hierarchical clustering and partial least square discriminant analyses (PLS-DA) previous normalization by sum, log-transform and autoscaling of the raw data (Supplementary figure 5).

**Supplementary figure 5**. a) Partial least squares-discriminate analysis (PLS-DA) of metabolic profiles from Critical (C), Severe (S), Mild (M) COVID-19 patients and Quality Control (P). PLS-DA according to metabolic profiles of Critical vs Mild b), Critical vs Severe d) and Mild vs Severe f) comparisons and their respective VIP values c), e), and g).


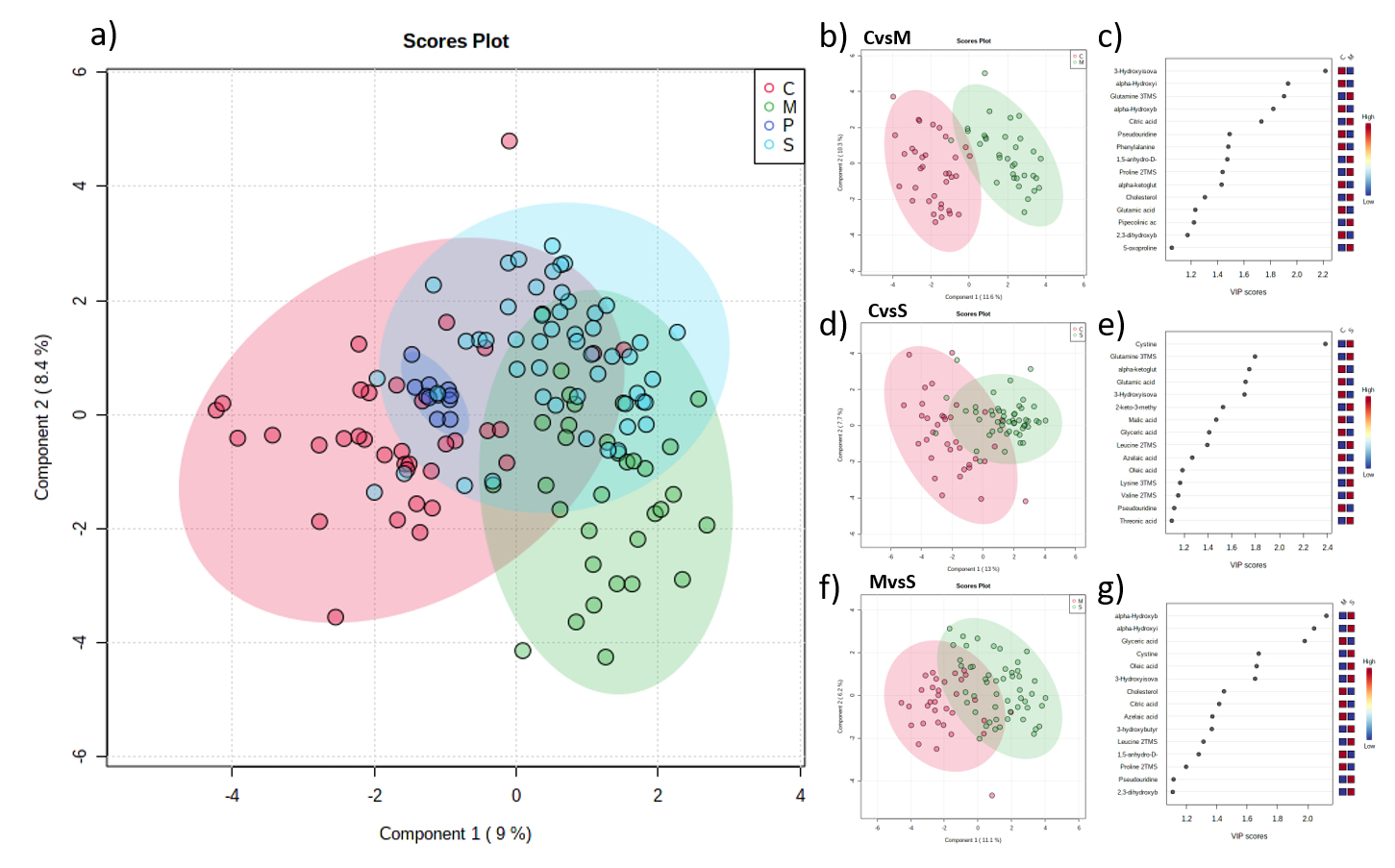


**Supplementary table 4.** Univariate analysis for the construction of the COVID-19 severity prediction model.

| **Variable** | **X^2^** | **p-value** |
| --- | --- | --- |
| Demographic features | | |
| Female (%) | 5.6998 | 0.01697 |
| Male (%) | 5.6998 | 0.01697 |
| Age (years) | 25.209 | 5.144×10^-7^ |
| Obesity | 7.1016 | 0.007702 |
| Diabetes Mellitus | 11.559 | 0.0006742 |
| Hypertension | 5.5488 | 0.01849 |
| Comorbidities (Number) | 18.3 | 1.887×10^−5^ |
| **Clinical features** | | |
| Body mass index(kg/m^2^) | 8.2383 | 0.004102 |
| Heart rate (beats per minute) | 16.279 | 5.467×10^−5^ |
| **Laboratory parameters** | | |
| Total monocytes (cells/mm^3^) | 4.8082 | 0.02832 |
| NT/LT ratio | 18.682 | 1.544×10^−5^ |
| Hemoglobin (g/dL) | 22.127 | 2.552×10^−6^ |
| Platelets (cells/mm^3^) | 6.7244 | 0.00951 |
| Glucose (mg/dL) | 10.814 | 0.001007 |
| Blood Urea Nitrogen (mg/dL) | 17.14 | 3.473×10^−5^ |
| Sodium (mmol/L) | 5.2666 | 0.02174 |
| Alanine aminotransferase (U/L) | 7.0625 | 0.007872 |
| Alkaline phosphatase (U/L) | 8.6006 | 0.00336 |
| Albumin (g/dL) | 48.075 | 4.103×10^−12^ |
| C reactive protein (mg/dL) | 8.101 | 6.717×10^−10^ |
| Ferritin (ng/dL) | 14.624 | 0.0001312 |
| Troponin (pg/mL) | 7.4755 | 0.006254 |
| Lactic dehydrogenase (U/L) | 40.061 | 2.462×10^−10^ |
| Creatinin phosphokinase (U/L) | 12.206 | 0.0004762 |
| D Dimer (ng/mL) | 4.9805 | 0.02563 |
| Fibrinogen (mg/dL) | 30.799 | 2.861×10^−8^ |
| **Arterial blood gases** | | |
| Anion gap (mmol/L) | 4.0371 | 0.1328 |
| **B lymphocytes subsets** | | |
| ASC (cells/µl) | 4.9945 | 0.02543 |
| CD27- (cells/µl) | 6.0823 | 0.01365 |
| Mature (cells/µl) | 6.9793 | 0.008246 |
| DN (cells/µl) | 4.8463 | 0.0277 |
| DN2 (cells/µl) | 4.1497 | 0.04164 |
| DN3 (cells/µl) | 6.5782 | 0.01032 |
| Naïve (cells/µl) | 5.8271 | 0.01578 |
| resN (cells/µl) | 5.8929 | 0.0152 |
| **T lymphocytes subsets** | | |
| CD8TEM (cells/µl) | 16.79 | 4.175×10^−5^ |
| TH1 (cells/µl) | 16.915 | 3.91×10^−5^ |
| Th17 (cells/µl) | 7.0562 | 0.007899 |
| Tc1 (cells/µl) | 27.258 | 1.781×10^−7^ |
| Tc17 (cells/µl) | 9.4158 | 0.002151 |
| **Low density granulocytes subsets** | | |
| LDG (cells/µl) | 21.24 | 4.051×10^−6^ |
| **Coagulation Pathway** | | |
| C Protein (%) | 5.0145 | 0.02514 |
| Von Willebrand Factor (%) | 4.0064 | 0.04533 |
| **Muscular atrophy** | | |
| Trim63 (pg/mL) | 35.08 | 3.165×10^−9^ |
| **Neutrophil extracellular traps** | | |
| Nets (ODI) | 6.0623 | 0.01381 |
| **Metabolomic profile** | | |
| α-Hydroxybutyric acid (AU) | 12.732 | 0.0003594 |
| α-Hydroxyisovaleric acid (AU) | 15.066 | 0.0001038 |
| 3-Hydroxyisovaleric acid (AU) | 24.427 | 7.718×10^−7^ |
| Glycerol (AU) | 20.045 | 12.082 |
| Proline 2TMS (AU) | 15.012 | 0.0001068 |
| Pipecolinic acid (AU) | 4.8573 | 0.02753 |
| 2,3-dihydroxybutanoic acid (AU) | 10.296 | 0.001334 |
| Threonine 3TMS (AU) | 3.9016 | 0.04824 |
| 3,4-dihydroxybutanoic acid (AU) | 4.7598 | 0.02913 |
| Malic acid (AU) | 6.1026 | 0.0135 |
| 5-oxoproline (AU) | 6.3927 | 0.01146 |
| Alpha-ketoglutarate (AU) | 7.2299 | 0.00717 |
| Glutamic acid 3TMS (AU) | 12.734 | 0.000359 |
| Phenylalanine 2TMS (AU) | 10.762 | 0.001036 |
| Glutamine 3TMS (AU) | 26.819 | 2.235×10^−7^ |
| Citric acid (AU) | 11.818 | 0.0005867 |
| Myristic acid (AU) |  |  |
| 1,5-anhydro-D-sorbitol(AU) | 20.612 | 5.624×10^−6^ |
| Myo-inositol (AU) | 8.4766 | 0.003598 |
| Cystine (AU) | 4.8124 | 0.02826 |
| Pseudouridine (AU) | 9.3611 | 0.002216 |
| Cholesterol (AU) | 11.069 | 0.0008778 |
| Cytokines and chemokines | | |
| IL-1RA (pg/mL) | 3.9418 | 0.0471 |
| IL-3 (pg/mL) | 5.4171 | 0.01994 |
| IL-6 (pg/mL) | 3.849 | 0.04978 |
| IL-8 (pg/mL) | 6.9824 | 0.008232 |
| IL 18 (pg/mL) | 12.103 | 0.0005034 |
| TNF-A (pg/mL) | 8.3586 | 0.003839 |
| VEGF (pg/mL) | 7.8584 | 0.005059 |
| MCP-1/CCL2 (pg/mL) | 9.7999 | 0.001745 |
| IP-10/CXCL10 (pg/mL) | 12.82 | 0.0003429 |

**Supplementary table 5**. Discrimination and calibration measures. Optimism index corrected obtained by bootstrapping 1000 samples of the original data. E_max_ = maximum difference in predicted vs calibrated probabilities.

| **Index name** | **Index original** | **Training** | **Test** | **Optimism-corrected** | **Optimism** |
| --- | --- | --- | --- | --- | --- |
| 𝜌 | 0.898 | 0.907 | 0.889 | 0.880 | 0.018 |
| Somer's D | 0.932 | 0.862 | 0.930 | 0.999 | -0.068 |
| R^2^ | 0.884 | 0.910 | 0.860 | 0.834 | 0.050 |
| AUC (C-statistic) | 0.966 | 0.932 | 0.965 | 0.999 | -0.033 |
| E_max_ | 0.000 | 0.000 | 0.108 | 0.108 | 0.108 |

**Supplementary figure 6.** Calibration plot for COVID-19 Severity Risk model obtained by bootstrapping 1000 samples of the original data (121 patients)


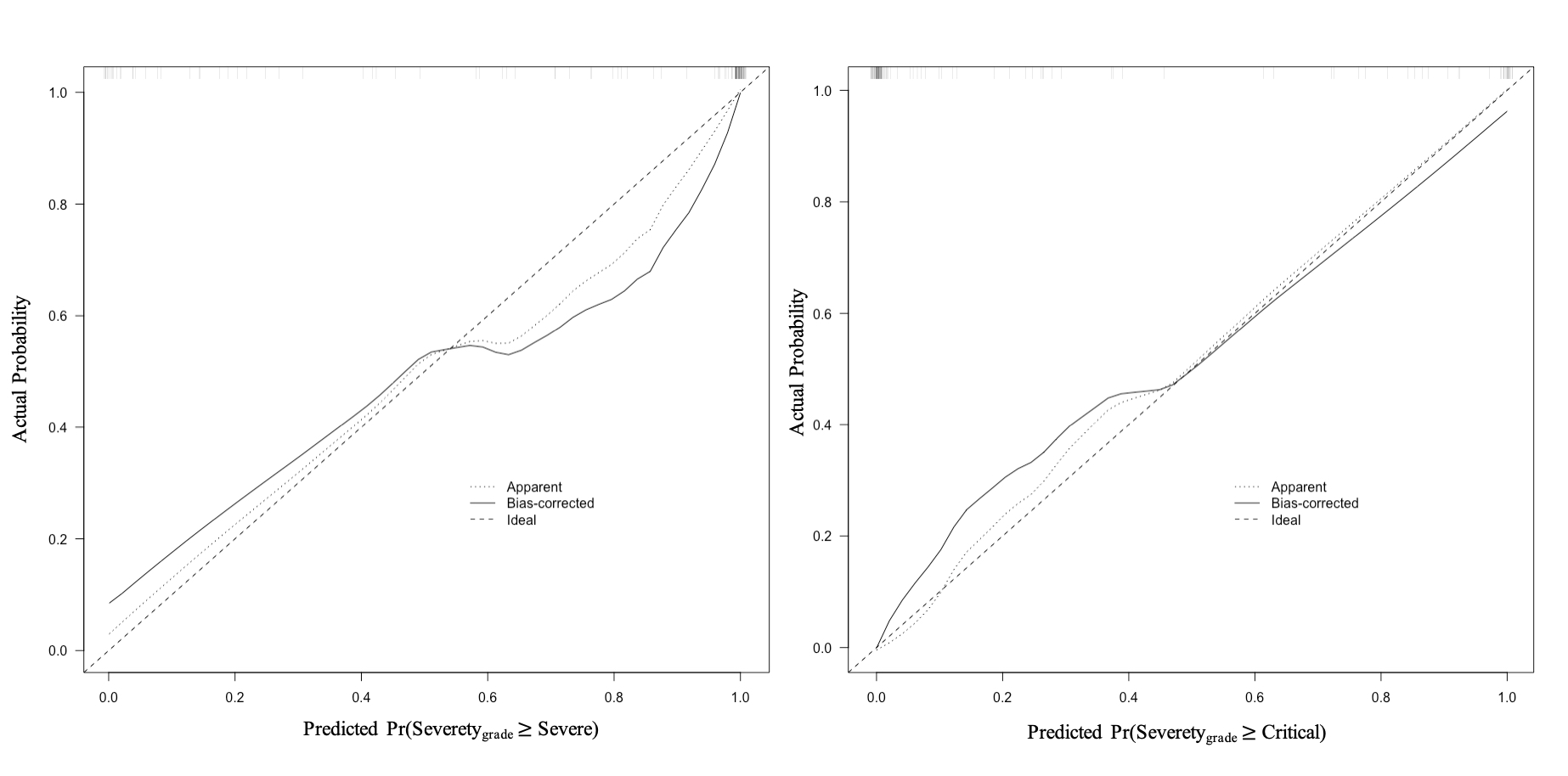


**References:**

1. Fiehn O. Metabolomics by Gas Chromatography-Mass Spectrometry: Combined Targeted and Untargeted Profiling. *Curr Protoc Mol Biol*. Apr 1 2016;114:30 4 1-30 4 32. doi:10.1002/0471142727.mb3004s114
